# Structural and Functional Insights into the Malaria Parasite Moving Junction Complex

**DOI:** 10.1371/journal.ppat.1002755

**Published:** 2012-06-21

**Authors:** Brigitte Vulliez-Le Normand, Michelle L. Tonkin, Mauld H. Lamarque, Susann Langer, Sylviane Hoos, Magali Roques, Frederick A. Saul, Bart W. Faber, Graham A. Bentley, Martin J. Boulanger, Maryse Lebrun

**Affiliations:** 1 Unité d'Immunologie Structurale, Institut Pasteur, Paris, France; 2 URA 2185 CNRS, Paris, France; 3 Department of Biochemistry & Microbiology, University of Victoria, Victoria, British Columbia, Canada; 4 UMR 5235 CNRS, Université de Montpellier 2, Montpellier, France; 5 Plate-Forme de Biophysique des Macromolécules et de leurs Interactions, Institut Pasteur, Paris, France; 6 Department of Parasitology, Biomedical Primate Research Centre, Rijswijk, The Netherlands; University of Texas Southwestern Medical Center, United States of America

## Abstract

Members of the phylum Apicomplexa, which include the malaria parasite *Plasmodium*, share many features in their invasion mechanism in spite of their diverse host cell specificities and life cycle characteristics. The formation of a moving junction (MJ) between the membranes of the invading apicomplexan parasite and the host cell is common to these intracellular pathogens. The MJ contains two key parasite components: the surface protein Apical Membrane Antigen 1 (AMA1) and its receptor, the Rhoptry Neck Protein (RON) complex, which is targeted to the host cell membrane during invasion. In particular, RON2, a transmembrane component of the RON complex, interacts directly with AMA1. Here, we report the crystal structure of AMA1 from *Plasmodium falciparum* in complex with a peptide derived from the extracellular region of *Pf*RON2, highlighting clear specificities of the *P. falciparum* RON2-AMA1 interaction. The receptor-binding site of *Pf*AMA1 comprises the hydrophobic groove and a region that becomes exposed by displacement of the flexible Domain II loop. Mutations of key contact residues of *Pf*RON2 and *Pf*AMA1 abrogate binding between the recombinant proteins. Although *Pf*RON2 contacts some polymorphic residues, binding studies with *Pf*AMA1 from different strains show that these have little effect on affinity. Moreover, we demonstrate that the *Pf*RON2 peptide inhibits erythrocyte invasion by *P. falciparum* merozoites and that this strong inhibitory potency is not affected by AMA1 polymorphisms. In parallel, we have determined the crystal structure of *Pf*AMA1 in complex with the invasion-inhibitory peptide R1 derived by phage display, revealing an unexpected structural mimicry of the *Pf*RON2 peptide. These results identify the key residues governing the interactions between AMA1 and RON2 in *P. falciparum* and suggest novel approaches to antimalarial therapeutics.

## Author Summary

Malaria arises from infection of erythrocytes by single-cell parasites belonging to the genus *Plasmodium*, the species *P. falciparum* causing the most severe forms of the disease. The formation of a moving junction (MJ) between the membranes of the parasite and its host cell is essential for invasion. Two important components of the MJ are Apical Membrane Antigen 1 (AMA1) on the parasite surface and the *Plasmodium* rhoptry neck (RON) protein complex that is translocated to the erythrocyte membrane during invasion. The extra-cellular region of RON2, a component of this complex, interacts with AMA1, providing a bridge between the parasite and its host cell that is crucial for successful invasion. The parasite thus provides its own receptor for AMA1 and accordingly this critical interaction is not subject to evasive adaptations by the host. We present atomic details of the interaction of *Pf*AMA1 with the carboxy-terminal region of RON2 and shed light on structural adaptations by each apicomplexan parasite to maintain an interaction so crucial for invasion. The structure of the RON2 ligand bound to AMA1 thus provides an ideal basis for drug design as such molecules may be refractory to the development of drug resistance in *P. falciparum*.

## Introduction

*Plasmodium spp*., and *P. falciparum* in particular, are devastating global pathogens that place nearly half the human population at risk to malaria, leading to more than 250 million cases yearly and over one million deaths [1]. The success of the malaria parasite can be attributed to its intracellular lifestyle, invading host cells both in liver and blood stages. Invasion of red blood cells is an active process involving a moving junction (MJ), which is formed by intimate contact between erythrocyte and parasite membranes and is thought to be coupled to the parasite's actin-myosin motor [2], [3]. A number of merozoite antigens, either exposed on the surface or stored in secretory organelles, play a role in the invasion process [4]. One of these is Apical Membrane Antigen 1 (AMA1), a type-one transmembrane protein secreted from the micronemes to the merozoite surface and present at the MJ [5], [6]. AMA1 is highly conserved in the *Plasmodium* genus [6] and, moreover, in the Apicomplexa phylum to which *Plasmodium* belongs [7], [8], suggesting a common functional role in diverse host cell invasion scenarios. In the apicomplexan organism *Toxoplasma gondii*, the receptor for AMA1 was shown to be Rhoptry Neck Protein 2 (RON2), a component of the parasite-derived RON protein complex that is secreted into the host cell during invasion and integrated into the host cell membrane [9], [10]. This interaction was subsequently confirmed in *P. falciparum* as well [11], [12]. Apicomplexans thus provide both receptor and ligand to drive active invasion.

In many malaria-endemic regions, *P. falciparum* has become resistant to classic drugs, such as chloroquine, and is rapidly developing resistance to recently introduced drugs. Since both AMA1 and RON2 are specific to Apicomplexa and essential for invasion, interruption of the AMA1-RON2 interaction presents an ideal new target for the design and development of inhibitors. This is supported by the recent observation that the invasion-inhibitory peptide R1 [13], [14] blocks interaction between AMA1 and the RON complex in *P. falciparum* [15], but due to the polymorphism of AMA1, the effectiveness of this peptide inhibitor is limited to a subset of parasite isolates. Interestingly, R1 does not prevent apical contact but no formation of a functional MJ ensues from this event [15].

Crystal structures of *Pf*AMA1 in complex with invasion-inhibitory antibodies [16], [17] have implicated a hydrophobic groove on Domain I (DI) of *Pf*AMA1 as being critical for function. The topological nature of the *Pf*AMA1 groove [18] is conserved in *P. vivax* AMA1 [19] and *T. gondii* AMA1 [20], and contains a number of residues that are conserved or semi-conserved across *Plasmodium* species, as well as other members of Apicomplexa [21], suggesting that it contributes to the receptor-binding site of AMA1. This was recently confirmed by the crystal structure of *Tg*AMA1 in complex with a synthetic peptide, *Tg*RON2sp, which inserts in the groove of *Tg*AMA1 [22].

Here, we report the crystal structure of the complex formed between *Pf*AMA1 and peptide segments of *Pf*RON2, which, together with our previous structural results on the *Tg*AMA1-*Tg*RON2 co-structure [22], highlights a conserved, crucial interaction in apicomplexan host cell invasion. Functional characterization of hot-spot residues driving AMA1-RON2 complex formation leads to a deeper understanding of key interactions occurring at the MJ of *P. falciparum* and reveals the molecular basis of cross-strain reactivity while preserving specificity for the species. We also describe the crystal structure of *Pf*AMA1 in complex with the invasion-inhibitory peptide R1 [14], and show that this peptide presents an intriguing structural mimicry of *Pf*RON2. Collectively, our results provide an important structural basis for designing cross-strain reactive molecules that inhibit invasion by *P. falciparum*.

## Results

### *Pf*RON2sp specifically binds to *Pf*AMA1

**Figure 1:**
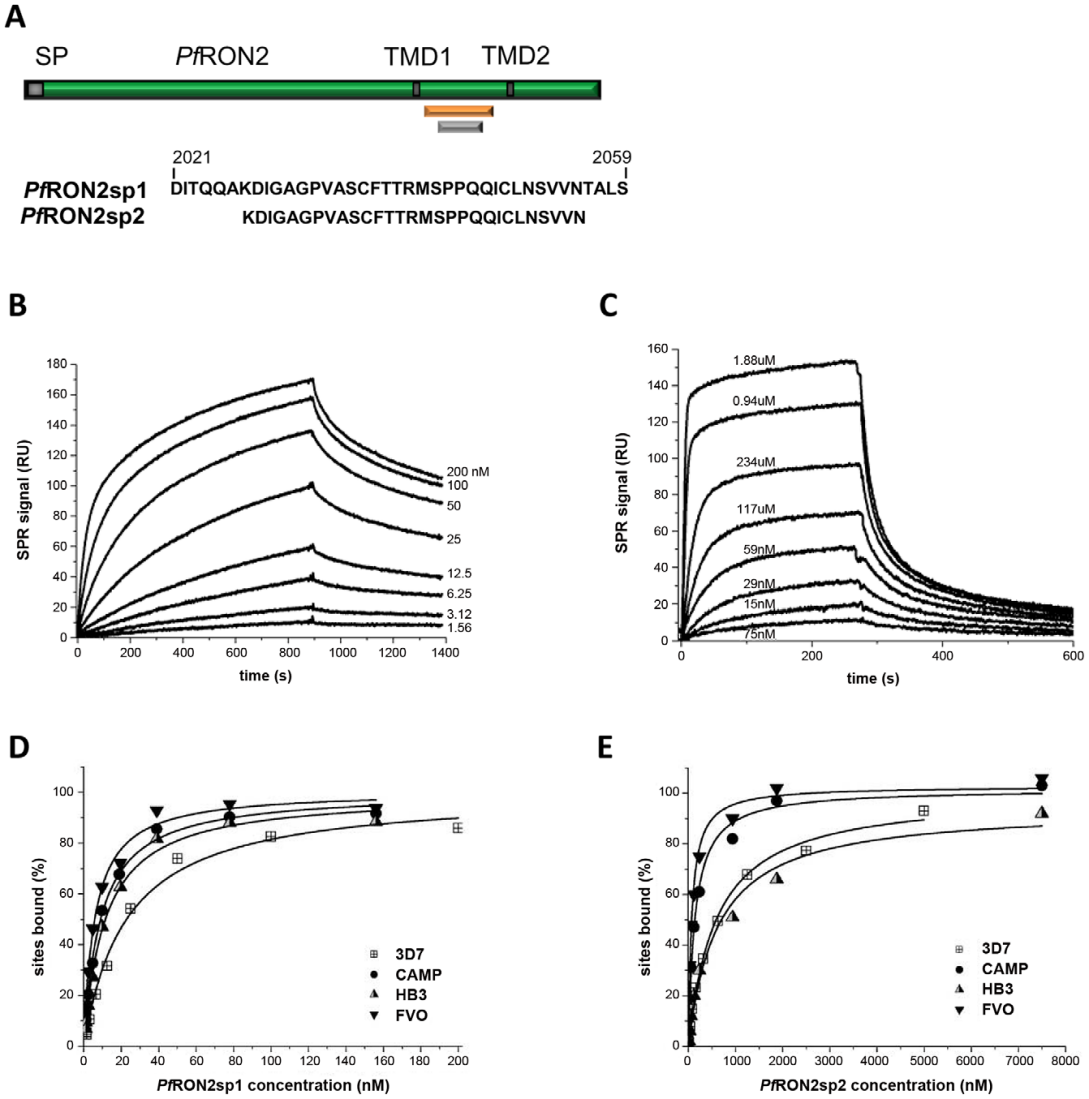
Surface Plasmon Resonance studies of peptides *Pf*RON2sp1 and *Pf*RON2sp2 binding to recombinant *Pf*AMA1 from multiple strains reveal that *Pf*RON2sp1 has a consistently higher affinity. (A) *Pf*RON2sp1 (orange) and *Pf*RON2sp2 (grey) represent peptides of *Pf*RON2 (green). SP, signal peptide. TMD, putative transmembrane domain. (B). Sensorgrams showing *Pf*RON2sp1 (analyte) binding to *Pf*AMA1 3D7 (immobilized). The *Pf*RON2sp1 concentrations are indicated for each curve (nM). (C). Sensorgrams showing *Pf*RON2sp2 (analyte) binding to *Pf*AMA1 CAMP (immobilized), with *Pf*RON2sp2 concentrations indicated. (D, E). Variation percentage of bound sites (deduced from the steady-state response) with respect to analyte concentration (D, *Pf*RON2sp1; E, *Pf*RON2sp2) obtained from binding to immobilized recombinant *Pf*AMA1 from strains 3D7 (shown in B), CAMP (shown in C), FVO and HB3. The derived apparent equilibrium dissociation constants K_D_ are given in Table 1.

From the 67-residue construct, *Pf*RON2-5, that we previously showed to have affinity for *Pf*AMA1 [11], and guided by the *Tg*AMA1-*Tg*RON2sp structure [22], we synthesized two analogous *Pf*RON2 peptides: *Pf*RON2sp1 (residues 2021–2059; numbering from the initiation methionine in PF14_0495), and *Pf*RON2sp2 (residues 2027–2055). Significantly, there is no polymorphism in this sequence among *P. falciparum* isolates. Both constructs incorporate a disulfide-bound β-hairpin loop proposed to be critical in complex formation [22] while *Pf*RON2sp2 is truncated at both the N- and C-termini (Fig. 1A). Since the extracellular region of *Pf*RON2 is non-polymorphic, we determined the affinity of both peptides for *Pf*AMA1 by Surface Plasmon Resonance (SPR) measurements using the 3D7, CAMP, FVO and HB3 proteins to explore the possible effects of AMA1 polymorphisms. The affinity of *Pf*AMA1 from 3D7 for *Pf*RON2sp1 is 25-fold higher than for *Pf*RON2sp2 (Fig. 1B to E, Table 1), highlighting a moderate, yet influential, role for the N- and C-terminal tails. Interestingly, K_D_ values for the *Pf*RON2sp peptides showed no significant variation in binding to *Pf*AMA1 from the four strains.

**Table 1:** Apparent equilibrium dissociation constants K_D_ (nM) for the binding of peptides *Pf*RON2sp1 and *Pf*RON2sp2 to AMA1 from different strains of *P. falciparum*.

Strain	*Pf*RON2sp1	*Pf*RON2sp2
3D7	20.3±6.3	520±74
CAMP	14.6±3.8	165±42
FVO	9.2±3.0	80±15
HB3	18.3±4.6	680±180

Independent experiments were performed at least three times and the values represent the mean ± SD.

**Figure 2:**
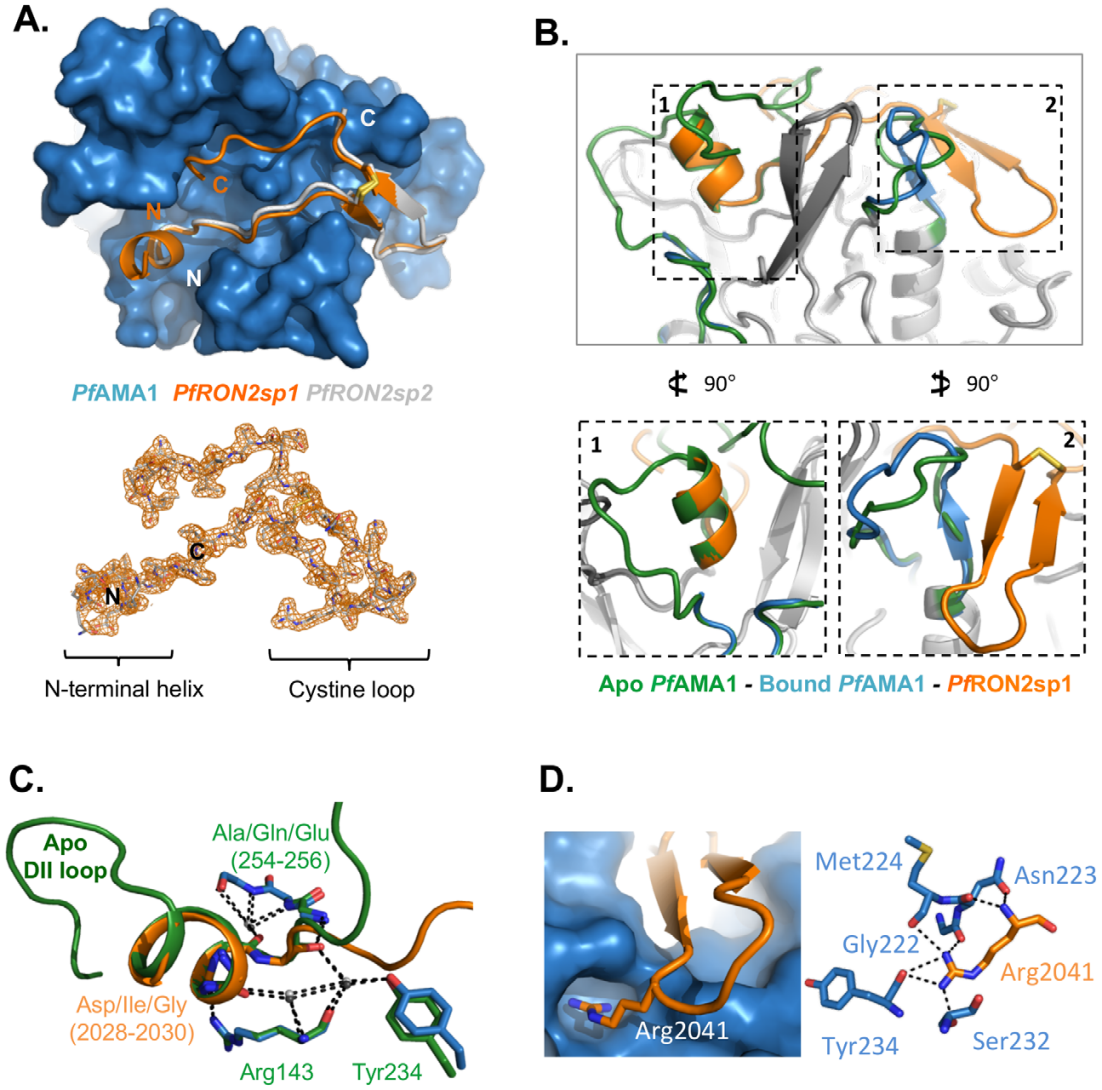
Structure of *Pf*AMA1 complexed with *Pf*RON2-derived peptides. (A) Top - Co-crystal structures of *Pf*AMA1 (blue surface) with *Pf*RON2sp1 (orange) and *Pf*RON2sp2 (grey), show a disulfide-anchored U-shaped conformation in the apical groove of *Pf*AMA1. Bottom - Electron density map (orange) for *Pf*RON2sp1 contoured at 1.0 σ, highlighting well ordered density from the N-terminal helix, through the cystine loop, to the C-terminal coil. (B) Notable changes in the structure of *Pf*AMA1 between the apo structure (green; PDB ID 1Z40) and the *Pf*AMA1-*Pf*RON2sp1 co-structure (blue-orange) as observed from a side view. Box 1 - The DII loop of apo *Pf*AMA1 is ejected from the apical groove during binding to *Pf*RON2sp1, leaving room for the *Pf*RON2sp1 N-terminal helix to occupy the space vacated by the DII loop helix. Box 2 - The β-strands of the *Pf*RON2sp1 cystine loop order a *Pf*AMA1 surface loop, generating a contiguous three-stranded β-sheet. (C) In the region of the *Pf*RON2sp1 N-terminal helix, there is notable structural mimicry to the *Pf*AMA1 apo DII loop, including several conserved residues, and a conserved hydrogen bonding network incorporating three buried water molecules. (D) Arg2041, specific to *P. falciparum*, fits snugly into a deep pocket in the surface of *Pf*AMA1 and is stabilized through a complex network of seven hydrogen bonds.

*Pf*RON2sp1 and *Pf*RON2sp2 were co-crystallized with the first two ectoplasmic domains (DI, DII) of recombinant *Pf*AMA1 3D7 or CAMP strains, respectively. The co-structure of *Pf*AMA1 3D7 *Pf*RON2sp1 (PDB entry code 3ZWZ) was refined to 2.2 Å resolution, while *Pf*AMA1 CAMP *Pf*RON2sp2 (PDB entry code 3SRI) was refined to 1.6 Å resolution (Tables 2, 3). The two co-structures overlay with a root mean square deviation (rmsd) of 0.81 Å in 304 Cα positions, and the two peptides alone overlay with a rmsd of 0.34 Å over the complete length of the modeled *Pf*RON2sp2 (25 Cα) (Fig. 2A). These data confirm that the reduced affinity of *Pf*RON2sp2 is due to the truncated N- and C-termini. Since *Pf*RON2sp1 is more biologically relevant than its truncated counterpart, it is used for the following analyses unless otherwise noted.

**Table 2:** Crystallographic parameters, data collection statistics and refinement summary.

	*Pf*AMA1 3D7-*Pf*RON2sp1	*Pf*AMA1 CAMP -*Pf*RON2sp2	*Pf*AMA1 3D7-R1
Spacegroup	P2_1_	P2_1_	P2_1_2_1_2_1_
a, b, c (Å)	70.15, 38.26, 70.75	70.72, 38.14, 72.08	38.32, 144.32, 145.64
α, β, γ (deg.)	90, 99.73, 90	90, 97.72, 90	90, 90, 90
Wavelength (Å)	0.9795	0.9537	0.9791
Resolution range (Å)	45.41-2.10	46.97-1.60	40.28-2.15
	(2.21-2.10)	(1.69-1.60)	(2.25-2.15)
Measured reflections	109520	153050	156625
Unique reflections	22041	48207	42798
Redundancy	5.0 (5.0)	3.2 (3.2)	3.7 (2.5)
Completeness (%)	100.0 (100.0)	94.9 (92.8)	95.3 (75.7)
*I/σ(i)*	8.7 (3.2)	12.7 (1.7)	13.3 (2.2)
R_merge_	0.140 (0.470)	0.056 (0.618)	0.075 (0.485)

Values in parenthesis are for the last resolution shell.

**Table 3:** Refinement statistics.

	*Pf*AMA1 3D7-*Pf*RON2sp1	*Pf*AMA1 CAMP-*Pf*RON2sp2	*Pf*AMA1 3D7-R1
Resolution (Å)	34.87–2.10 (2.15-2.10)	35.04-1.60 (1.64-1.60)	37.06-2.15 (2.15-2.21)
R_cryst_/R_free_	0.164/0.201 (0.202/0.241)	0.176/0.195 (0.230/0.247)	0.171/0.214 (0.215/0.249)
No. of atoms			
Protein A/B/C/D/E/F	2377/259	2309/190	2375/2385/157/60/135/77
Solvent	226	265	450
Glycerol	30	N/A	N/A
B-values (Å^2^)			
Protein A/B/C/D/E/F	17.3/29.9	27.5/48.3	36.4/40.5/50.6/77.8/61.5/92.6
Solvent	28.7	37.6	46.1
Glycerol	39.1	N/A	N/A
r.m.s. deviation from ideality			
Bond lengths (Å)	0.015	0.010	0.010
Bond angles (deg.)	1.52	1.05	1.10
Ramachandran statistics			
Most favoured	97.6%	96.7%	96.3%
Allowed	2.4%	3.3%	3.7%
Disallowed	0.0%	0.0%	0.0%

*Pf*RON2sp1, traced from Thr2023 to Leu2058, includes a disulfide bridge between Cys2037 and Cys2049 and makes several direct contacts with *Pf*AMA1 (Fig. S1), resulting in a total buried surface area of 3154 Å^2^ (1441 Å^2^ for *Pf*AMA1 and 1713 Å^2^ for *Pf*RON2sp1). Overall, the binding paradigm established by *Tg*AMA1-*Tg*RON2sp [22] is maintained, with an N-terminal helix seated at one end of the AMA1 receptor-binding groove and extended through an ordered coil to a disulfide-closed β-hairpin loop, generating a U-shaped conformation (Fig. 2A). Similarly, exposing a functional receptor-binding groove on AMA1 requires displacement of the extended non-polymorphic DII loop, which adopts a disordered state (not modeled between Lys351 to Ala387); this region is stabilized by DI in apo *Pf*AMA1 (Fig. 2B). Intriguingly, the backbone of the N-terminal helix and additional coil of *Pf*RON2sp1 (2024-QQAKDIGAG-2032) overlays remarkably well with a section of the apo *Pf*AMA1 DII loop (360-YEKIKEGFK-368) (rmsd<0.4 Å), which also includes a helical region (Fig. 2B - box 1). Three water molecules buried by the DII loop in the apo form are retained in the receptor-bound state and facilitate a network of hydrogen bonds that bridge *Pf*AMA1 DI to either the DII loop or *Pf*RON2sp in apo *Pf*AMA1 or the receptor complex, respectively (Fig. 2C). The majority of intermolecular contacts are formed by the segment Lys2027-Met2042 of *Pf*RON2sp1. An influential residue on *Pf*RON2 appears to be Arg2041, a residue specific to the *P. falciparum* species, located at the tip of the β-hairpin with its guanidyl group fitting snugly into a preformed pocket of *Pf*AMA1 (Fig. 2D).

### R1 occupies the *Pf*RON2sp-binding site on *Pf*AMA1

**Figure 3:**
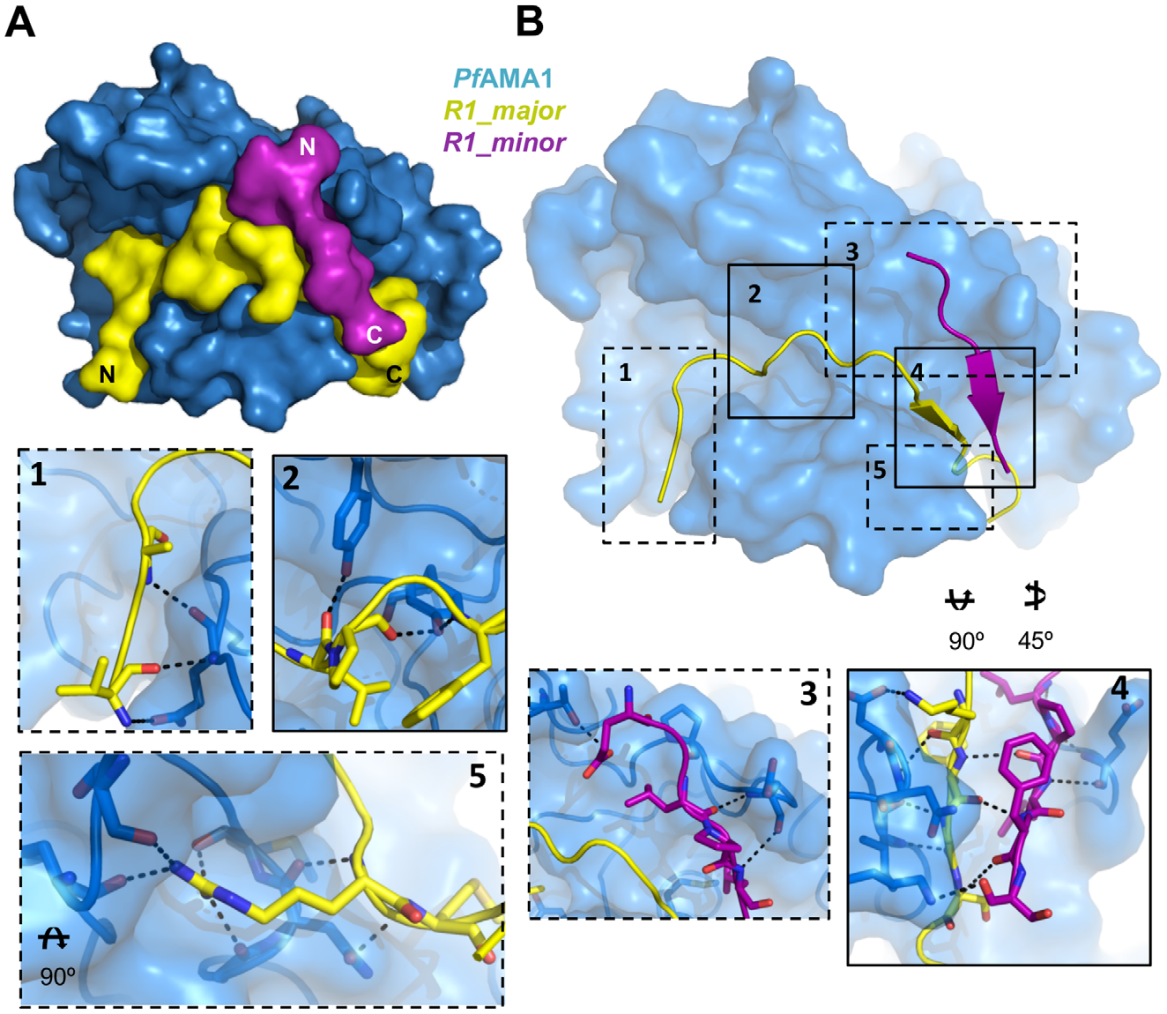
Structure of *Pf*AMA1 complexed with R1 peptide. (A). The co-crystal structure of *Pf*AMA1 (blue surface) with R1 reveals two bound peptides, R1 major (yellow) and R1 minor (purple). (B). Detailed analysis of interactions at the *Pf*AMA1–R1-major, *Pf*AMA1–R1-minor, and R1-major–R1-minor interfaces. Surface representation of *Pf*AMA1 (blue), with R1-major (yellow) and R1-minor (purple) shown as cartoons. Box 1 – R1-major anchors its N-terminus to *Pf*AMA1 through 3 backbone hydrogen bonds. Box 2 – the central region of the *Pf*AMA1 apical groove is occupied by R1-major through both hydrophobic and polar interactions. Box 3 – R1-minor forms most of its anchor points to *Pf*AMA1 through the apical loops and does not contact the base of the groove, which is occupied by R1-major. Panel 4 – Backbone hydrogen bonds between R1-minor and R1-major generate a β-sheet, while R1-major is further pinned to the *Pf*AMA1 groove through 3 hydrogen bonds. Panel 5 – R1-major integrates into *Pf*AMA1 with the use of an arginine knob-in-hole interaction stabilized by 6 hydrogen bonds, which is also exploited by*Pf*RON2sp.

The invasion-inhibitory peptide R1, comprising 20 residues (VFAEFLPLFSKFGSRMHILK) [14], has been shown by nuclear magnetic resonance (NMR) to bind to the *Pf*AMA1 hydrophobic groove, but this study gave little structural detail of the interaction [15]. We therefore crystallized *Pf*AMA1 3D7 (DI and II) with R1 to compare with the *Pf*RON2 complex. Surprisingly, two molecules of R1 are bound to *Pf*AMA1, which we denote respectively as the major peptide (R1-major), lying deeply in the binding groove, and the minor peptide (R1-minor), lying above R1-major and making fewer contacts with *Pf*AMA1 (Fig. 3 and Table S1). Several solvent molecules bridge directly between *Pf*AMA1 and R1-major. As in the *Pf*AMA1-*Pf*RON2sp complexes, the N-terminus of R1-major binds to a region of *Pf*AMA1 that becomes exposed after displacement of the DII loop.

R1-major makes several direct contacts with *Pf*AMA1 (113 interatomic distances<3.8 Å), including 19 hydrogen bonds and a salt bridge between the amino group of Lys-P11 (R1 peptide residues numbers are prefixed by P) and the Asp227 carboxylate group of *Pf*AMA1 (Table S1A). Contacts made by R1-minor to *Pf*AMA1 are fewer (26 contacts<3.8 Å) and include only five hydrogen bonds (Table S1B). Interactions between R1-major and R1-minor are maintained by a total of 24 interatomic contacts, including three hydrogen bonds (Table S1C). In total, 3025 Å^2^ of molecular surface is buried between *Pf*AMA1 and the two peptides, with R1-major contributing about 75% to this area. The buried surface between R1-major and R1-minor is 563 Å^2^, reflecting the smaller number of close interatomic contacts between these two components.

Since the structure of the *Pf*AMA1 3D7-R1 complex revealed two bound peptide molecules, binding measurements of R1 to *Pf*AMA1 3D7 were made by isothermal titration calorimetry (ITC) to examine the stoichiometry (Fig. S2). The measured K_D_ of 145 nM is comparable with previous measurements by SPR [13] and the deduced stoichiometry was 1∶1 over the peptide concentrations used. This implies that the second binding site in the crystal structure (R-minor) has an affinity that could not be determined under the experimental conditions used for ITC but can be estimated to be at least 10-fold weaker than the major site.

### R1 mimicry of *Pf*RON2

**Figure 4:**
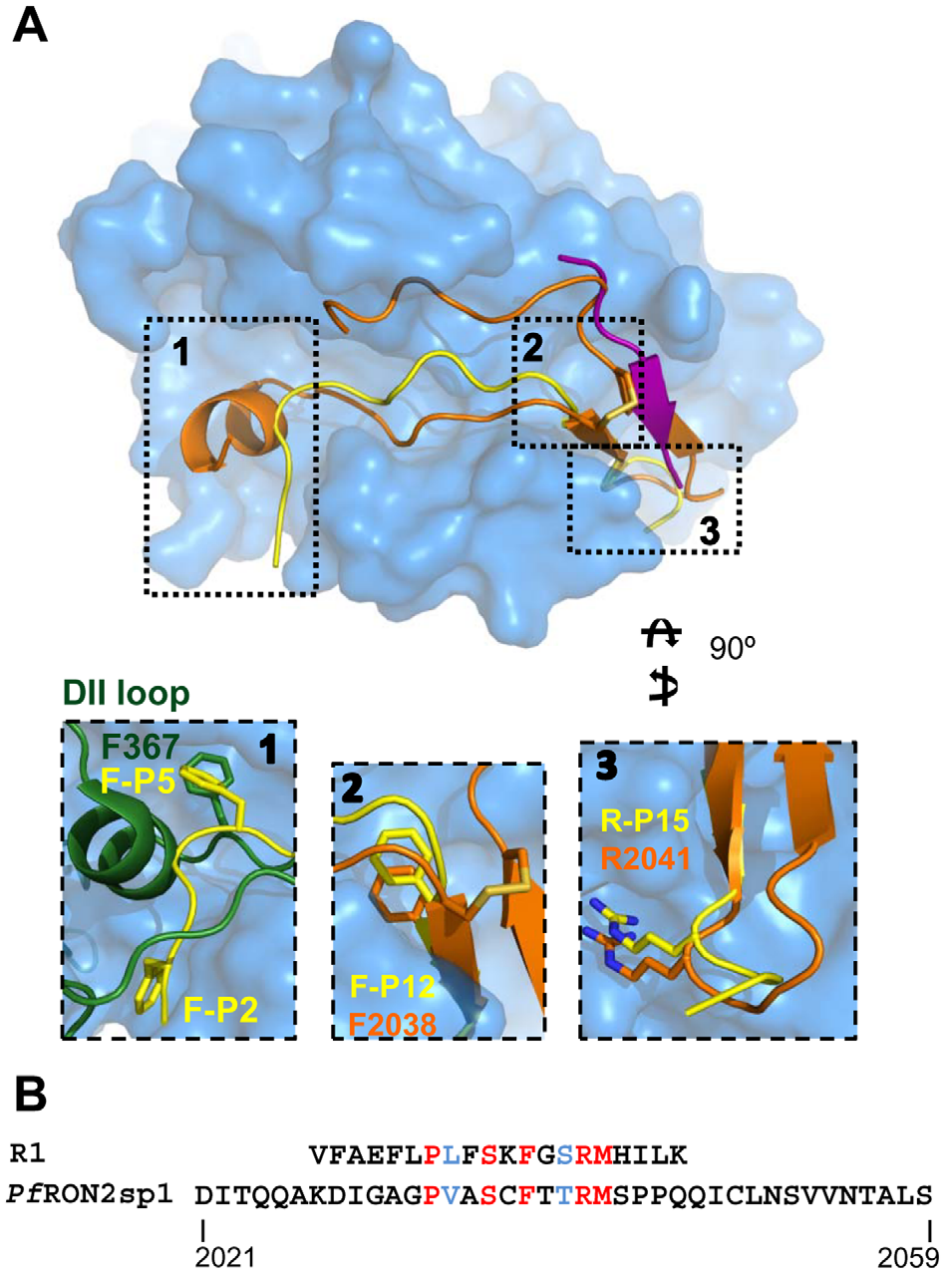
Structural mimicry of *Pf*RON2 by peptide R1 in binding to *Pf*AMA1. (A) Top (left) and end-on (right) views of *Pf*AMA1-*Pf*RON2sp1 (orange cartoon) overlayed on *Pf*AMA1-R1-major (yellow)/R1-minor (purple), show that the *Pf*AMA1 groove is capable of accepting only *Pf*RON2sp1 or the two R1 peptides at one time. Box 1 shows that Phe-P5 of R1 mimics Phe367 of the DII loop, while boxes 2 and 3 highlight spatial conservation of a phenylalanine anchor at the center of the groove, and a knob-in-hole interaction incorporating the peptide Arg-P15. R1-major is shown in yellow, *Pf*RON2sp1 in orange and apo PfAMA1 in green. (B). Comparison of the R1 and *Pf*RON2sp1 sequences reveals five identical (red) and two similar (blue) residues.

While R1-major follows the general contour of the receptor-binding groove, it does so in a linear rather than the U-shaped conformation adopted by *Pf*RON2sp1 (Fig. 4A). R1-minor occupies a similar region in space as the second strand of the *Pf*RON2sp β-hairpin, contacting the same DI loop of *Pf*AMA1 but running in the opposite direction to form a parallel two-stranded β-sheet with the major peptide (Fig. 4A). Portions of R1-major exhibit structural similarity to *Pf*RON2, displaying a 1.2 Å rmsd in the twelve Cα positions (*Pf*RON2sp1, Ala2031 to Met2042; R1-major, Phe-P5 to Met-P16) (Fig. 4A). Moreover, sequence alignment based on the structural superposition reveals a remarkable similarity between the central regions of the two ligands; the segments Ala2031-Met2042 of *Pf*RON2 and Phe-P5–Met-P16 of R1 have five identical amino acids and two conservative differences (Fig. 4B). R1-major residue Arg-P15 contributes the most contacts to *Pf*AMA1 and is positioned within the same pocket of *Pf*AMA1 as *Pf*RON2 Arg2041 (Fig. 4A - box 3) where it maintains six of the seven hydrogen bonds observed for *Pf*AMA1-*Pf*RON2sp. Interestingly, while *Pf*RON2 mimicry is observed in the cystine loop-binding region (Phe2038/Phe-P12 to Arg2041/Arg-P15), R1-major establishes clear anchor points in the hydrophobic groove different from *Pf*RON2; Phe-P2 and Phe-P5 brace the peptide N-terminus in the region exposed by displacement of the DII loop, with Phe-P5 occupying the pocket left vacant by Phe367 of *Pf*AMA1 (Fig. 4A - box 1).

### *Pf*AMA1 Polymorphisms at positions 175 and 225 are determinant for the 3D7 specificity of R1

**Figure 5:**
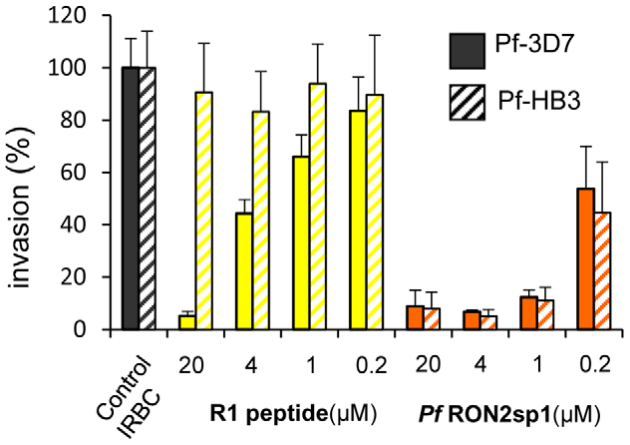
Highly potent cross-strain inhibition of red blood cell invasion of *Pf*RON2sp1. Comparison of *Pf*RON2sp1 and R1 peptides (concentrations 0.2 to 20 µM) in inhibiting red blood cell invasion by *P. falciparum* 3D7 or HB3 highlights the higher inhibitory efficiency and cross-strain reactivity of *Pf*RON2sp1. Parasitemia of control infected red blood cells (IRBC) 16 hours post-invasion was used as the 100% invasion reference. Means (± SD for N = 3) are shown.

R1 is strain specific, binding to *Pf*AMA1 from the 3D7 (cognate antigen) and D10 strains, but with much reduced affinity to the HB3 or W2mef proteins, as determined by ELISA [14] or SPR [13] measurements (recapitulated in Table S2). In contrast, *Pf*RON2sp1 bound to all the *Pf*AMA1 proteins tested (Table 1) with a higher affinity than for R1 peptide. Consistent with these values, *Pf*RON2sp1 displayed a higher capacity to inhibit red cell invasion by *P. falciparum* 3D7 than the R1 peptide (Fig. 5). Moreover, *Pf*RON2sp1 shows cross-strain inhibition of invasion as expected from its biological function (Table 1), contrasting with the more restricted strain specificity of R1 (Fig. 5, Table S2) [14].

**Figure 6:**
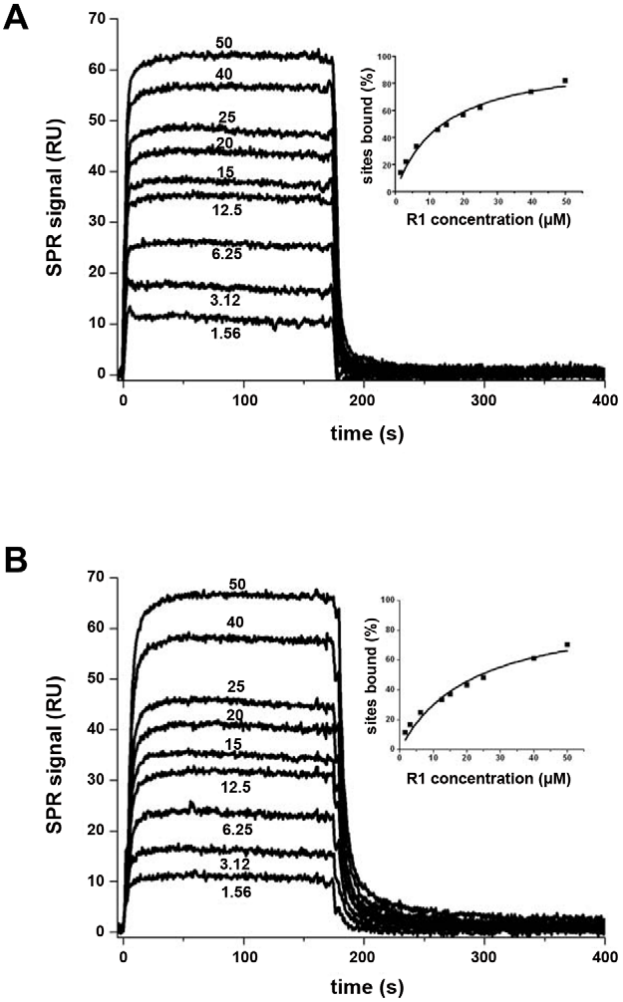
Surface Plasmon Resonance studies of peptide R1 binding to *Pf*AMA1 mutants 3D7mut and Dico3. (A). Left - sensorgrams, showing R1 (analyte) binding to *Pf*AMA1 3D7mut (immobilized). R1 concentrations are indicated for each curve (µM). Right - the variation in percentage of bound sites (deduced from the steady-state response) with respect to analyte concentration. (B). Left - sensorgrams, showing R1 (analyte) binding to Dico3 (immobilized), with R1 concentrations indicated. Right - the variation in percentage of bound sites (deduced from the steady-state response) with respect to analyte concentration. The equilibrium dissociation constant K_D_ derived from the steady state binding curves is 15.2 µM for 3D7mut and 22.3 µM for Dico3.

The *Pf*AMA1 3D7-R1 crystal structure shows that three polymorphic residues (175, 224 and 225) contact R1-major (Table S2). The 224 polymorphism, Met/Leu, is conservative and since contacts are formed by the main chain only, this should not affect R1 specificity. The 3D7 and D10 antigens both carry Tyr175 and Ile225; for the W2mef and HB3 antigens, residue 175 is Tyr and Asp, respectively, and residue 225 is Asn in both. Thus, polymorphisms at positions 225 and possibly 175 appear to be determinant for the 3D7 specificity of R1 at the major peptide-binding site (Table S2A). R1-minor contacts polymorphic residue 230, which is Lys in all strains studied (Table S2B). As our data suggest a weak affinity for this binding site, however, it is unlikely that this polymorphism has a significant effect on the specificity for R1. We examined these polymorphisms further using the mutant *Pf*AMA1 Dico3 [23], which differs only at residue 175 for the 3D7-contacting residues (Table S2A), and a 3D7 mutant with the substitution Ile225Asp, which we call 3D7mut. The equilibrium K_D_, determined from the SPR steady-state responses to R1 binding, was 15.2±1.9 µM for 3D7mut and 22.3±3.3 µM for Dico3, showing a reduction in affinity of over 200-fold with respect to the native 3D7 antigen (Fig. 6, Table S2C). This affinity is comparable to that observed for HB3 and W2mef [13] (recapitulated in Table S2), and confirms that both Tyr175 and Ile225 are important for the strain-specific recognition of R1. Tyr175, located at the tip of a flexible DI loop that is solvent-exposed in the apo antigen [18], becomes buried by R1-major and forms a hydrogen bond to this ligand *via* the phenol group. Ile225 is also buried by R1-major, forming a pair of hydrogen bond *via* its main chain to the R1-major main chain.

### Hot spots driving specific *Pf*AMA1-*Pf*RON2 complex formation

**Figure 7:**
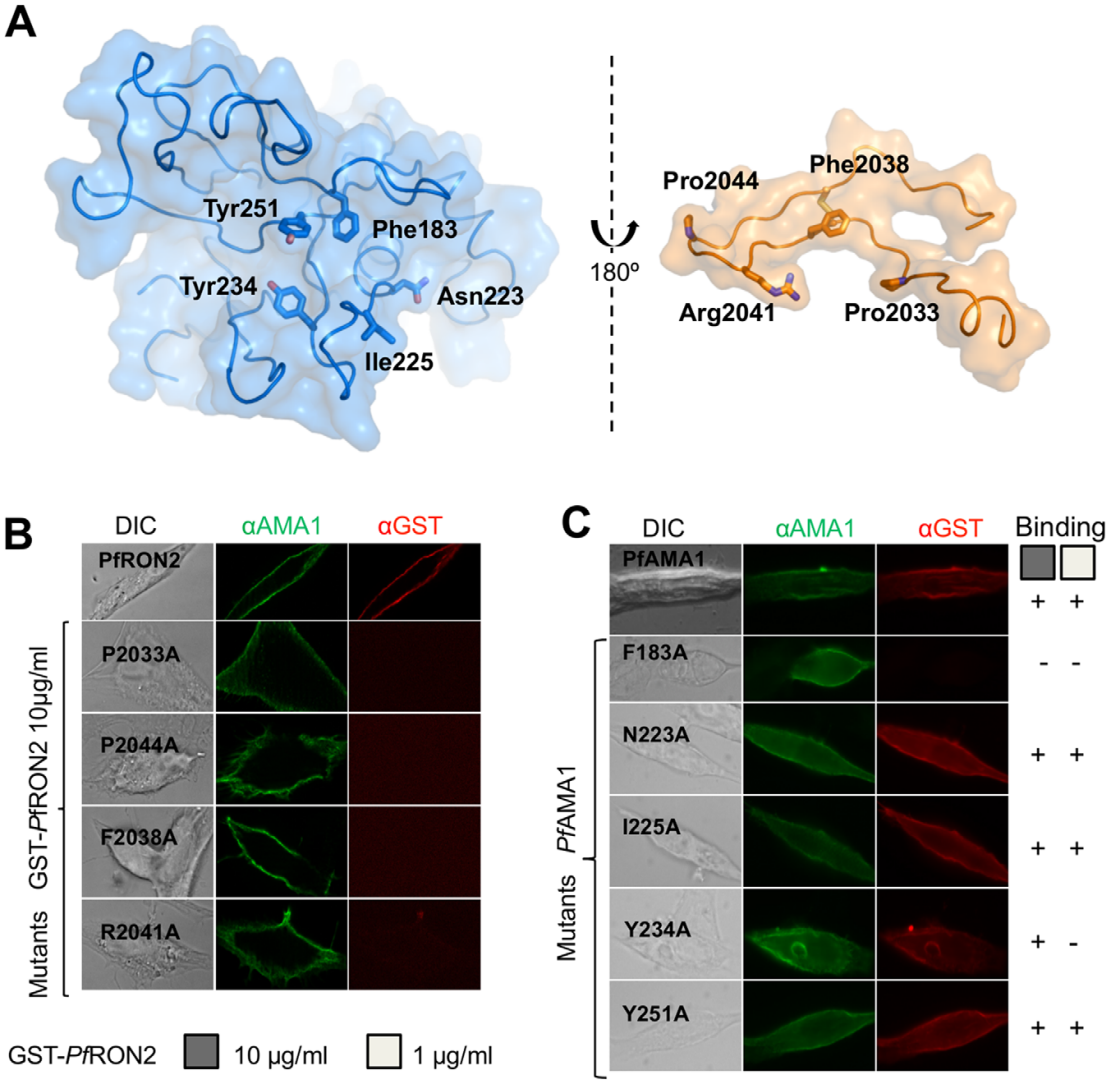
Mutations of *Pf*AMA1 and *Pf*RON2-5 reveal residues critical for high affinity interaction. (A) Interface between *Pf*AMA1 and *Pf*RON2sp1 shown in open-book presentation. Residues of both components that were mutated are labeled. (B). Binding characteristics of recombinant GST-*Pf*RON2-5 mutants to dissect hot-spot residues in *Pf*RON2. *Pf*AMA1-expressing BHK-21 cells were incubated with 10 µg/ml of *Pf*RON2 or mutated proteins (GST-fusion proteins), washed and the binding of recombinant *Pf*RON2 fragment was revealed with anti-GST antibody. *Pf*AMA1 was detected with mAb F8.12.19, which recognizes extracellular Domain III. (C). Binding consequences of *Pf*AMA1 mutations. Mutated versions of *Pf*AMA1 were expressed on the surface of BHK-21 cells and incubated with wild-type *Pf*RON2 recombinant proteins at 10 and 1 µg/ml.

Guided by the similarities between the *Pf*RON2sp and R1 co-structures, and the conservation of key contact residues (Fig. 7A), we probed the functional importance of a subset of *Pf*RON2 residues by testing the binding to BHK-21 cells expressing *Pf*AMA1 of GST-*Pf*RON2-5 fusion proteins carrying single alanine mutations at: Pro2033 (aligns structurally with Pro7 of peptide R1, which was shown to be critical for binding [24]), Phe2038 (interacts with invariant residue Phe183 in the hydrophobic groove and aligns structurally with Phe12 of R1), Arg2041 (extensive contacts with *Pf*AMA1 and structurally equivalent to Arg-P15 of R1) and Pro2044 (the peptide bond Ser2043-Pro2044 is *cis* and is thus important for the β-hairpin conformation). Consistent with the structure, mutation of Arg2041 to Ala abrogated binding to *Pf*AMA1 (Fig. 7B). Similar effects were observed with Pro2044, Phe2038 and Pro2033 mutations, the latter also shown to be a key residue in the *Tg*AMA1-*Tg*RON2 interaction [22].

Similarly, a subset of key *Pf*AMA1 residues was also chosen for mutation: Phe183 (an invariant residue that contributes to the hydrophobic groove and that interacts with Phe2038 of *Pf*RON2 *via* aromatic interactions), Asn223 (which makes important polar interactions with *Pf*RON2), residue 225 (a polymorphic residue that contributes many contacts to *Pf*RON2 in the structure both the CAMP (Asn225) and 3D7 (Ile225) complexes), Tyr234 (which makes polar contacts to Arg2041 of *Pf*RON2) and Tyr251 (which has been suggested by previous studies to be important [12], [25]). A clear role for Phe183 in the *Pf*AMA1-*Pf*RON2 complex formation was evident when expressed on the surface of BHK-21 cells and tested for their ability to bind GST-*Pf*RON2-5 fusion protein (Fig. 7C). A less pronounced role of Tyr234 was observed and none for the remaining residues, including Tyr251. Although these conclusions differ from those of others [12], [25], these results are consistent with the limited contacts shown by this residue in the structures and with our earlier findings on the *Tg*AMA1-*Tg*RON2 interaction, where the equivalent *Tg*AMA1 residue, Tyr230, had a minimal effect on the binding.

## Discussion

The structure of *Pf*AMA1 in complex with the extracellular region of its receptor *Pf*RON2 and the accompanying functional analysis reveal atomic details of the interaction between two key partners at the MJ. The binding site on *Pf*AMA1 includes the hydrophobic groove and a region that becomes exposed by displacement of the flexible DII loop from its apo conformation. Comparison of residues from both components at the *Pf*AMA1-*Pf*RON2 interface with those of other apicomplexan homologs underscores the separate co-evolution of the receptor-ligand pair in members of the phylum.

The DII loop displays a strong propensity for mobility in *P. falciparum* [16], [18] and *P. vivax* AMA1 structures [19], particularly at its N- and C-terminal extremities (weak or absent electron density); the central region of the DII loop is more structured and stabilized by contacts with DI, and is better defined in some of these AMA1 structures. Here, we show that the DII loop is displaced by *Pf*RON2sp, as well as by the R1 peptide. In *T. gondii*, the DII loop is 14 residues shorter than in the *Plasmodium* orthologs and appears less mobile [20] but nonetheless is readily displaced by *Tg*RON2sp [22]. Flexibility may therefore have an important functional role: it protects a significant portion of the binding site in apo AMA1 against the host's immune response but can be readily displaced to extend the hydrophobic groove for effective binding to RON2. The anti-*Pf*AMA1 invasion-inhibitory monoclonal antibody 4G2, which binds to the N- and C-termini of the DII loop [19], probably prevents its displacement for effective binding to *Pf*RON2. The absence of polymorphisms in the DII loop in spite of immune targeting of this region underlines its important functional role [21].

**Figure 8:**
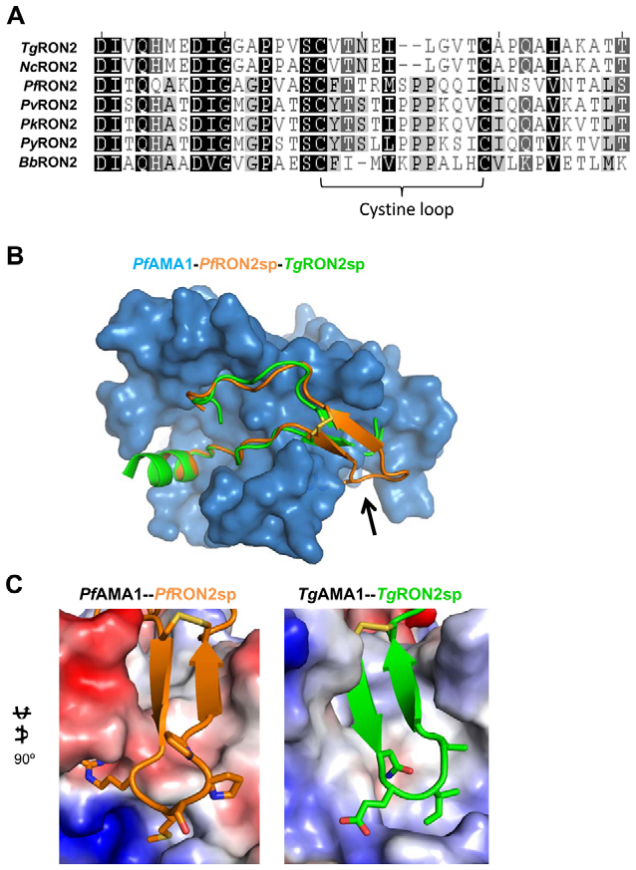
The RON2 cystine loop governs specificity. (A). Alignment of RON2 sequences truncated to correlate *Pf*RON2sp1 with RON2 sequences from the following accession numbers: *Tg*RON2 - TGME49_100100, *Nc*RON2 - NCLIV_064620, *Pf*RON2 - PF14_0495, *Pv*RON2 - PVX_117880, *Py*RON2 – PY_06813, *Bb*RON2 (BBOV_I001630). (B). Overlay of *Tg*RON2sp (green; PDB ID 2Y8T) onto *Pf*AMA1-*Pf*RON2sp (blue-orange) shows that both peptides adopt a helix/coil/cystine loop/coil architecture in the AMA1 groove, with the highest divergence localized to the cystine loop (black arrow). (C). Electrostatic surface renderings of *Pf*AMA1 (left) and *Tg*AMA1 (right), with the secondary structure of the RON2 binding partner and residues defining the base of cystine loop shown, illustrates that both interactions are highly complementary, but highly genus specific.

**Figure 9:**
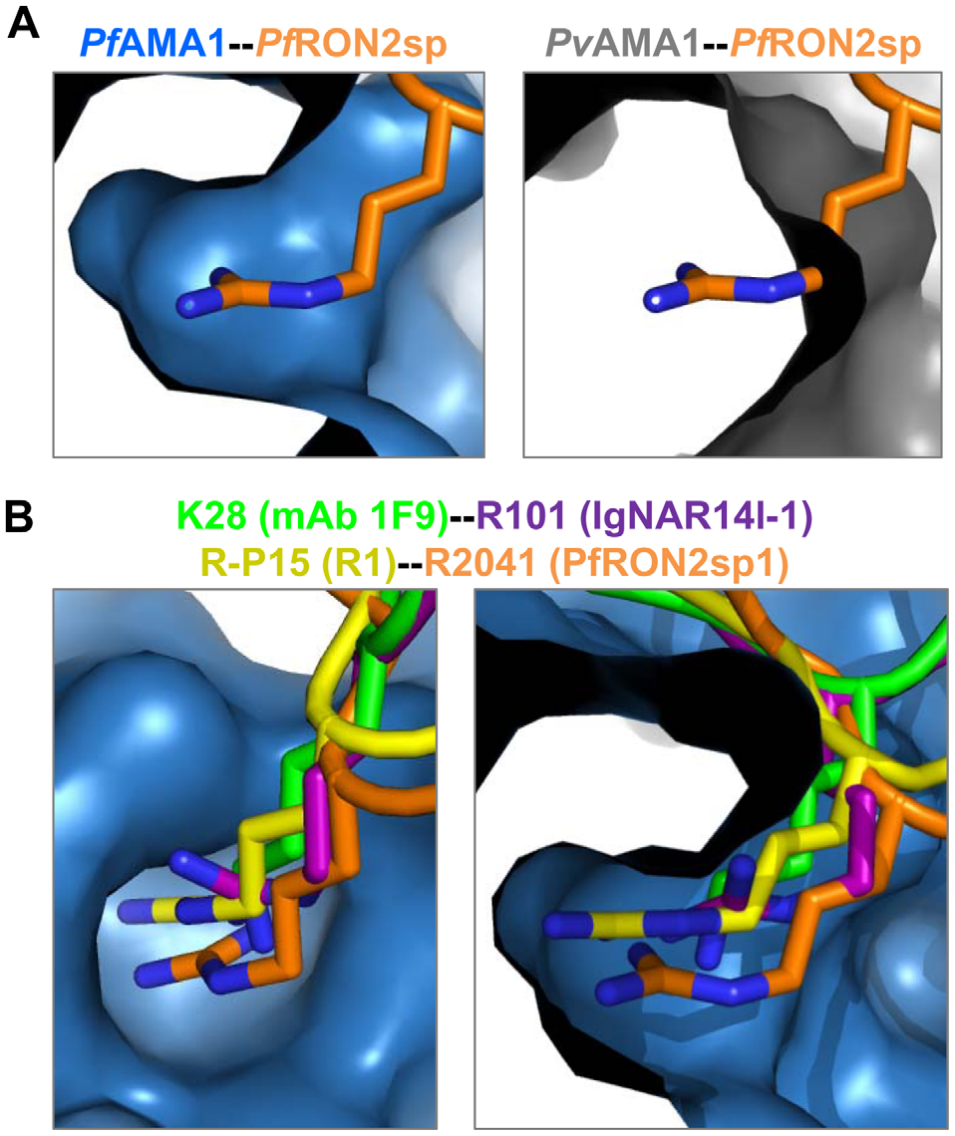
The Arg knob-in-hole interaction is critical for species selectivity and interaction with invasion inhibitory antibodies and peptides. (A). Left - A cut-away surface of *Pf*AMA1 (blue), reveals that Arg2041 of *Pf*RON2sp1 (orange) integrates deeply into a well-defined pocket. Right - However, no analogous pocket is observed in *Pv*AMA1 (grey; PDB ID 1W8K). (B). Peptides and antibodies known to be invasion inhibitory for *P. falciparum* occupy the key Arg binding site, as shown by orthogonal views of the *Pf*AMA1-*Pf*RON2sp1 co-structure (blue-orange) overlayed with the mAb 1F9 co-structure (1F9, green; PDB ID 2Q8B), IgNAR14l-1 co-structure (IgNAR, purple; PDB ID 2Z8V), and R1 co-structure (R1, yellow; reported here).

We have previously demonstrated an evolutionary constraint on the AMA1–RON2 interaction within apicomplexan parasites [11]. Our functional analysis of the *Tg*AMA1-*Tg*RON2sp co-structure suggested that the cystine loop initially anchors the receptor to the hydrophobic groove, causing expulsion of the DII loop to promote interaction throughout the entire binding site [22]. Comparison of the *Tg*AMA1-*Tg*RON2sp and *Pf*AMA1-*Pf*RON2sp co-structures reveals that the cystine loop, while conserved across the two genera, is the most divergent region within the RON2 (Fig. 8). The separate co-evolution of the AMA1-RON2 pair in Apicomplexa is clearly illustrated by the difference between the cystine loop conformations of *Pf*RON2sp and *Tg*RON2sp. In particular, this allows Arg2041 to access the specific *Pf*AMA1 pocket (Fig. 8), where it participates in an intricate network of polar interactions. From mutagenesis, we have demonstrated a crucial role of Arg2041 in complex formation (Fig. 7B). Moreover, this region of the cystine loop also appears to play an influential role in species selectivity as superposition of *Pv*AMA1 structure [19] onto *Pf*AMA1-*Pf*RON2sp shows that Arg2041 would be sterically hindered at the interface but Thr, the equivalent residue in *Pv*RON2 from *P. vivax*, can be accommodated (Fig. 9A). This accounts for our prior observation that the original 67-residue segment of *Pf*RON2 does not bind to *Pv*AMA1 [11].

An additional feature of the *Pf*RON2sp cystine loop region is the presence of a *cis* peptide bond between Ser2043 and Pro2044; the Ser-Pro-Pro segment contributes negligible buried surface area but is important for maintaining the β-hairpin conformation for efficient complex formation. Sequence alignment reveals that the Pro duo (Pro2044–Pro2045) is preserved in all analyzed *Plasmodium* species (Fig. 8A) and is thus likely important for specific recognition of AMA1. We propose that it provides necessary internal structure at the tip of the cystine loop and places the disulfide bond in the proper orientation to brace the AMA1-RON2 interaction. The influential role of Pro2044 is confirmed by mutagenesis where substitution with Ala, which would disfavor the *cis* peptide bond, abrogates *Pf*AMA1*-Pf*RON2 binding (Fig. 7B). While *T. gondii* does not share the conserved proline pair, its cystine loop is two residues shorter (Fig. 8A), which mirrors the narrower groove of *Tg*AMA1. Altogether, the overall U-shape architecture of RON2 in complex with AMA1 appears to be remarkably well maintained within apicomplexan parasites but specific features are clearly visible in the cystine loop of *Pf*RON2 and *Tg*RON2, highlighting how a receptor-ligand complex has evolved to maintain a common and crucial event in the biology of these parasites.

Although the *Pf*AMA1-*Pf*RON2 interface is highly conserved, five polymorphic residues of *Pf*AMA1 contact the non-polymorphic *Pf*RON2sp [26]. Of these, however, only residue 225 (Asn/Ile) varies significantly. The remaining polymorphisms should not affect binding as they involve main chain contacts only (residues 172, 174, 187 and 224). Our study allows a detailed structural assessment of polymorphism at residue 225 since complexes with *Pf*AMA1 from the 3D7 (Ile225) and CAMP (Asn225) strains were determined. The 3D7 and CAMP orthologs both maintain two hydrogen bonds between the main chain of residue 225 and *Pf*RON2 Thr2039. However, Ile225 presents a deep pocket to Arg2041 with apolar contacts formed between the aliphatic regions of these two side chains, while Asn225 presents a shallower pocket to Arg2041 with the Asn225 amide group stacking against the guanidyl group. Nonetheless, our binding studies by SPR show no significant difference in the affinity of these two *Pf*AMA1 homologs for *Pf*RON2sp2. Sequence variations at *Pf*RON2-interacting positions, 172(Glu/Gly), 187(Glu/Asn) and 225 (Ile/Asn) are represented by the strains 3D7, CAMP, FVO and HB3 that we have analyzed by SPR; the very similar K_D_ constants, ranging from approximately 10 to 20 nM, confirm that these exert little effect in the strength of the interaction.

Peptide R1 shows a more restricted specificity as it binds strongly to the cognate 3D7 and closely related D10 antigens but only weakly to orthologs that do not carry the same polymorphic amino acids at position 175 or 225 (Table S2). Tyr175 in *Pf*AMA1 3D7 makes a hydrogen bond to the main chain of R1-major but, as this residue is located in a flexible loop with some freedom to adapt to the *Pf*AMA1-R1 interface, it is unclear why the Asp175 polymorphism leads to reduced affinity. In the case of Ile225 of *Pf*AMA1 3D7, the main chain forms two hydrogen bonds to the main chain of R1-major but the preference of R1 for the Ile225 polymorphism remains unexplained as it contrasts with *Pf*RON2sp where main chain hydrogen bonds are also formed by both Ile225 (3D7) and Asn225 (CAMP) to the main chain of *Pf*RON2. This emphasizes that specificity differences may present subtleties that are difficult to decipher. Here, the crystal structure of R1 in complex with the 3D7mut (Ile225Asn) and Dico3 (Tyr175Asp) mutants of *Pf*AMA1 would provide invaluable insights into this question. Taken together, these results highlight that unlike the natural ligand *Pf*RON2, R1, which was selected by phage display, is highly susceptible to polymorphisms.

R1 exhibits a close structural similarity to *Pf*RON2, with the major/minor peptide pair displaying a similar boomerang form as *Pf*RON2, binding to the same region of *Pf*AMA1 and following the same general contour of the binding-site groove. Our structural data show that binding of R1-minor is dependent upon prior binding of R1-major as it lies above the latter in the binding groove and makes fewer contacts to *Pf*AMA1. This, indeed, is consistent with the ITC measurements that show a stoichiometry of 1∶1, indicating a weaker affinity for the minor peptide-binding site. R1-major is thus favored as the principle inhibitor of the interaction with *Pf*RON2, but this does not preclude a contribution by the minor peptide-binding site at high peptide concentrations.

Therapeutic strategies aimed at inhibiting the interaction between *Pf*AMA1 and *Pf*RON2 should be very effective in treating malaria as they address a critical phase in the life cycle of the parasite and, importantly, should not be compromised by polymorphism since the *Pf*AMA1-*Pf*RON2 interface is highly conserved. Our results provide a structural basis for designing inhibitors against the most virulent malaria parasite. The *Pf*RON2sp1 peptide used in this study has a very high affinity to *Pf*AMA1 and is very efficient at inhibiting invasion. Moreover, in contrast to the less strongly binding peptide R1, *Pf*RON2sp1 is not strain specific. Structural details of the *Pf*AMA1-*Pf*RON2 interaction offer the possibility to design molecules with the desired specific inhibitory properties by *in silico* screening and structural validation. The binding of *Pf*RON2 Arg2041 to a specific pocket on *Pf*AMA1 could be a critical target region. Indeed, the important role played by Arg-P15 at the *Pf*AMA1-R1 interface closely mirrors the equivalent interaction in the *Pf*AMA1-*Pf*RON2sp complexes and, interestingly, the same pocket is occupied by Arg and Lys in *Pf*AMA1 complexes with the invasion inhibitory antibodies IgNAR [17] and 1F9 [16], respectively (Fig. 9B). Phe2038 (corresponding to Phe-P12 in R1) is also a key residue, as its substitution by Ala affected binding. The importance of this sub-site is further highlighted by the concomitant loss in affinity when Phe183 (with which it interacts) was mutated in *Pf*AMA1. Collectively, these data provide a firm basis for designing molecules with optimal inhibitory properties to treat malarial infection.

## Materials and Methods

### Recombinant protein production

(i) Baculovirus insect cell expression: A synthetic codon-optimized gene encoding DI and DII of *Pf*AMA1 3D7 [27] (residues 104–438; numbering based on the initiation methionine, PF11_0344) (GenScript) was subcloned into a modified pAcGP67B vector (Pharmingen) for expression in insect cells using established protocols [20]. Final yield of recombinant protein was approximately 3 mg per L of culture.

(ii) *P. pastoris* expression: Synthetic genes were optimized for *Pf*AMA1 coding of residues 97–442, from strains 3D7 (Genbank accession number U33274), CAMP (accession number M34552) and HB3 (accession number U33277). Potential N-glycosylation sites were mutated and genes were cloned EcoRI-KpnI in the pPicZalpha A vector (Invitrogen), resulting in an 11-residues sequence extension followed by myc-epitope and hexa-His tags at the C-terminus), expressed in *P. pastoris*, and purified as described [28]. Yield after purification was approximately 20 mg per L of culture. *Pf*AMA1 FVO (residues 25–545, no tags, accession number AJ277646) was produced as described before [29]. The DiCo3 protein was modified compared to the published protein [23]; it includes the *Pf*AMA1 FVO prodomain (amino acids 25–96) and one additional mutation to minimize proteolytic cleavage Lys376–>Arg (B. Faber, unpublished results). The *Pf*AMA1 3D7mut (Ile225–>Asn, residues 25–545, no tags) mutant was generated by site-directed mutagenesis (Genscript) and produced in *P. pastoris* in a similar fashion to the native protein [29].

### Peptide synthesis

A 39-residue peptide corresponding to residues 2021 to 2059 of *Pf*RON2 (*Pf*RON2sp1) was synthesized by Kinexus (Vancouver, Canada) and disulfide cyclized. Lyophilized *Pf*RON2sp1 was solubilized in 100% DMSO and subsequently diluted in HBS (20 mM HEPES pH 7.5, 150 mM NaCl) for use in co-crystallization and functional studies. Peptides *Pf*RON2sp2 (residues 2027 to 2054) and R1 were synthesized by PolyPeptide (Strasbourg, France) and solubilized in 3.5% DMSO for subsequent use.

### Crystallization and data collection

Crystals of *Pf*AMA1 3D7 *Pf*RON2sp1 were grown in 30% PEG400, 100 mM Tris-HCl pH 8.5, 200 mM tri-sodium citrate dihydrate and the protein (5 mg/mL final concentration) incubated with *Pf*RON2sp1 (1∶2 molar excess). A crystal in cryoprotectant buffer was flash cooled at 100 K and diffraction data were collected on beamline 9-2 at SSRL (Stanford Synchrotron Radiation Laboratory, Stanford, US). Crystals of *Pf*AMA1 CAMP *Pf*RON2sp2 were obtained in 20% PEG 4000, 0.1 M Tris/HCl pH 8.6, 0.1 M sodium acetate and 20% isopropanol and the protein (6.4 mg/mL final concentration) incubated with *Pf*RON2sp2 (1∶5 molar excess). Diffraction data were collected from a crystal in cryoprotectant buffer at 100 K on beamline ID29 at European Synchrotron Radiation Facility (Grenoble, France). Crystals of *Pf*AMA1 3D7 R1 were obtained in 15% PEG 4000, 0.1 M Tris/HCl pH 8.5, 0.1 M sodium acetate and 10% isopropanol and the protein (5.4 mg/mL final concentration) incubated with R1 (1∶6 molar excess). Diffraction data were collected at 100 K on beamline PROXIMA 1 at SOLEIL (St. Aubin, France).

### Data processing, structure solution and refinement

Diffraction data were processed using Imosflm [30] or XDS [31] and Scala [32] in the CCP4 suite of programs [33]. Crystallographic parameters and data collection statistics are given in Table 2. Initial phases were obtained by molecular replacement using PHASER [34] or AMoRe [35] with the unliganded *Pf*AMA1 structure (PDB 1Z40). Tracing of the *Pf*RON2 and R1 peptides, and addition of solvent molecules, was performed manually in COOT [36] and refinement was performed with Refmac5 [37] or *autoBUSTER* (Global Phasing Ltd, Cambridge, UK). A summary of refinement statistics is given in Table 3. All molecular representation figures were generated in the PyMOL Molecular Graphics System, version 1.2r3pre, Schrödinger, LLC. Coordinates and structure factors have been deposited in the Protein Data Bank with the following entry codes: *Pf*AMA1-*Pf*RON2sp1, 3ZWZ; *Pf*AMA1-*Pf*RON2sp2, 3SRI; *Pf*AMA1-R1, 3SRJ.

### Binding studies by SPR

SPR measurements were made with a Biacore 2000 instrument (Biacore AB). AMA1 proteins diluted in 10 mM sodium acetate pH 4.5 for 3D7, CAMP, HB3 and FVO strains, or pH 4.0 for 3D7mut and Dico3, were covalently immobilized by an amine-coupling procedure on CM5 sensor chips (GE Healthcare). The reference flow cell was prepared by the same procedure in absence of protein. Binding assays were performed at 25°C in PBS and 0.005% Tween 20 by injecting a series of peptide (*Pf*RON2sp1 and *Pf*RON2sp2 on 3D7, CAMP, HB3 and FVO, and R1 on 3D7mut and Dico3) concentrations at a constant flow rate of 5 µL/min. A heterologous peptide was used to verify the absence of non-specific binding. Peptide dissociation was realized by injecting the running buffer, and the surface was regenerated by injecting glycine/HCl pH 1.5 followed by SDS 0.05%. Control flow cell sensorgrams were subtracted from the ligand flow cell sensorgrams and averaged buffer injections were subtracted from analyte sensorgrams. For peptide R1, steady-state signals (Req) were obtained directly from the plateau region of the sensorgrams, while for *Pf*RON2sp peptides, estimated values of Req were obtained by extrapolation from the experimental curves since the association phase did not reach a final equilibrium state. All calculations were made using the BIAevaluation 4.2 software (BIAcore AB). The saturation curves obtained by plotting R_eq_ versus the peptide concentration were fitted with a steady-state model to obtain the R_max_ and the apparent equilibrium dissociation constants, K_D_. To normalize the response for the different ligands, these curves were reported as the percentage of bound sites (ratio R_eq_/R_max_) versus the analyte concentration..

#### Isothermal calorimetry

ITC measurements were made using a ITC_200_ calorimeter (MicroCal). *Pf*AMA1 3D7 (*P. pastoris*) and peptide R1 were diluted in PBS to final concentrations of 0.6 µM and 55 µM, respectively. *Pf*AMA1 3D7 (initial volume 200 µL) was titrated at 25°C by consecutive injections of the peptide R1 (2 µL aliquots at 3 min intervals). Raw data were normalized and corrected for the heat of dilution of R1 in PBS. Binding stoichiometry was determined by fitting the final data to a 1∶1 interaction model using the Origin7 software (OriginLab).

### *P. falciparum* cultures and invasion assays

The *P. falciparum* cell cultures and the invasion assays were performed as described previously [11]. Briefly, highly synchronized *P. falciparum* 3D7 and HB3 schizonts (1.5% hematocrit, 1.5% parasitemia) were incubated with R1 or *Pf*RON2sp1 peptides. Blood smears were collected 16 hours post-invasion and used for ring-stage parasites counting. The results presented are representative of three independent experiments, each performed in triplicate.

### Transient transfection experiments and cell binding assays

Cell binding assays using *Pf*AMA1-expressing BHK-21 cells and recombinant GST-*Pf*RON2-5 fusion proteins were performed as previously described [11]. Although not quantitative, this cell-binding assay truly reflects the interaction between AMA1 and RON2 as we carefully checked all the experimental steps as well as the image recording as described below. Transfections were carried out using Lipofectamine Reagent (Invitrogen) as instructed by the manufacturer with 3×10^5^ BHK-21 cells grown on coverslips for 24 h in 6 well plates. Cells were grown for an additional 24 h post-transfection before subsequent analysis. Expression and correct folding of *Pf*AMA1 (and the mutants) at the host cell surface was verified by IFA performed with or without permeabilisation, using antibodies either specific to the cytoplasmic tail (anti-myc tag) or specific to the extracellular ectodomain of *Pf*AMA1 (mouse mAb F8.12.19 [38]). For binding assays, coverslips from a same transfection experiment were washed in HBSS (Invitrogen) before addition of recombinant *Pf*RON2-5 wild type or mutants diluted in HBSS at 10, 1 or 0.1 µg/ml. Coverslips incubated with GST were systematically used as a control. After five washes in PBS to remove unbound protein, cells were fixed in 4% PAF and further processed for IFA as described above [11]. The binding characteristics of RON2 (anti-GST labelling) on the *Pf*AMA1 mutant were only considered valid when its signal was identical to that of wild type *Pf*AMA1. All other micrographs were obtained with a Zeiss Axiophot microscope equipped for epifluorescence. Adobe photoshop (Adobe Systems, Mountain View, CA) was used for image processing. Matching pairs of images were recorded with the same exposure time and processed identically.

The *Pf*AMA1 and GST-*Pf*RON2 mutated constructs were generated by site directed mutagenesis using Quickchange II XL protocol (Stratagene).

## Supporting Information

Figure S1 **Detailed analysis of interactions at the** ***Pf*** **AMA1-** ***Pf*** **RON2sp1 interface.** (A). Open-book surface representation of *Pf*AMA1 (left) and *Pf*RON2sp1 (right) showing the extensive involvement of residues from both molecules in forming a complex interface. Residues involved in hydrogen bonding are coloured blue, while residues contributing significant buried surface area (BSA>20 Å^2^ for *Pf*AMA1, >5 Å^2^ for *Pf*RON2sp1) are colored green. (B). Table of residues involved in hydrogen bonding at the *Pf*AMA1- *Pf*RON2sp1 interface (left) and residues contributing significant buried surface area (right), as calculated by PISA (http://www.ebi.ac.uk/msd-srv/prot_int/pistart.html). Polymorphic residues of *Pf*AMA1 are shown in blue.
(PPTX)

Figure S2 **Isothermal titration calorimetry of peptide R1 binding to** ***Pf*** **AMA1 3D7.**
(PPTX)

Table S1 **Polar interactions and buried surface areas in the** ***Pf*** **AMA1-R1 crystal structure.** (A). Polar contacts between *Pf*AMA1 3D7 and R1-major (column 1), and buried surface areas of individual residues of *Pf*AMA1 3D7 (column 2) and R1-major (column 3). Salt bridges are indicated in bold. (B). Polar contacts between *Pf*AMA1 3D7 and R1-minor (column 1), and buried surface areas of individual residues of *Pf*AMA1 3D7 (column 2) and R1-minor (column 3). (C). Polar contacts between R1-major and R1-minor (column 1), and buried surface areas of individual residues of R1-major (column 2) and R1-minor (column 3). Polymorphic residues of *Pf*AMA1 are shown in blue.
(PPTX)

Table S2 **Polymorphic residues of** ***Pf*** **AMA1 contacting peptide R1.** (A). Polymorphic residues contacting R1-major showing the sequence for strains analyzed using ELISA (*) [14], SPR (^+^) [13] and in this study using SPR (°). (B). Polymorphic residues contacting R1-minor, showing the sequence for strains as presented in (A). (C). Binding to *Pf*AMA1, classified as strong (s) or weak (w) for the studies presented in (A) and (B).
(PPTX)

Table S3 **Primers used in this study.**
(PPTX)
