# Supplementary material for: Structural and Functional Insights into the Malaria Parasite Moving Junction Complex
Source: PLoS Pathog. 2012 Jun 21;8(6):e1002755. doi: 10.1371/journal.ppat.1002755 (PMC3380929; doi:10.1371/journal.ppat.1002755)
Supplement: Figure S1 — Detailed analysis of interactions at the Pf AMA1- Pf RON2sp1 interface. (A). Open-book surface representation of PfAMA1 (left) and PfRON2sp1 (right) showing the extensive involvement of residues from both molecules in forming a complex interface. Residues involved in hydrogen bonding are coloured blue, while residues contributing significant buried surface area (BSA>20 Å2 for PfAMA1, >5 Å2 for PfRON2sp1) are colored green. (B). Table of residues involved in hydrogen bonding at the PfAMA1- PfRON2sp1 interface (left) and residues contributing significant buried surface area (right), as calculated by PISA (http://www.ebi.ac.uk/msd-srv/prot_int/pistart.html). Polymorphic residues of PfAMA1 are shown in blue. (PPTX) [file ppat.1002755.s001.pptx]

## Slide 1
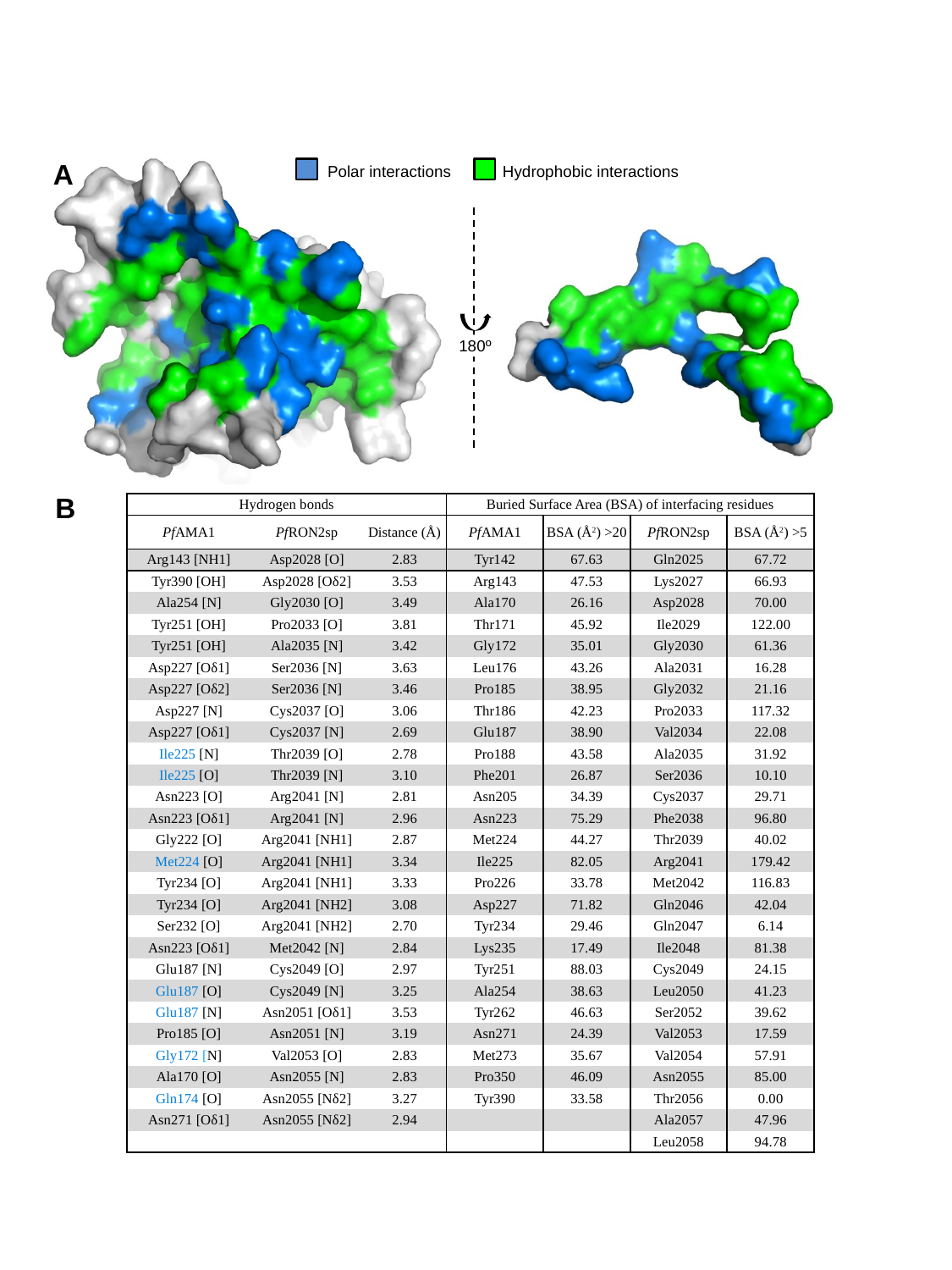

A
Polar interactions
Hydrophobic interactions
180º
B
| Hydrogen bonds | | | Buried Surface Area (BSA) of interfacing residues | | | |
| --- | --- | --- | --- | --- | --- | --- |
| PfAMA1 | PfRON2sp | Distance (Å) | PfAMA1 | BSA (Å2) >20 | PfRON2sp | BSA (Å2) >5 |
| Arg143 [NH1] | Asp2028 [O] | 2.83 | Tyr142 | 67.63 | Gln2025 | 67.72 |
| Tyr390 [OH] | Asp2028 [Oδ2] | 3.53 | Arg143 | 47.53 | Lys2027 | 66.93 |
| Ala254 [N] | Gly2030 [O] | 3.49 | Ala170 | 26.16 | Asp2028 | 70.00 |
| Tyr251 [OH] | Pro2033 [O] | 3.81 | Thr171 | 45.92 | Ile2029 | 122.00 |
| Tyr251 [OH] | Ala2035 [N] | 3.42 | Gly172 | 35.01 | Gly2030 | 61.36 |
| Asp227 [Oδ1] | Ser2036 [N] | 3.63 | Leu176 | 43.26 | Ala2031 | 16.28 |
| Asp227 [Oδ2] | Ser2036 [N] | 3.46 | Pro185 | 38.95 | Gly2032 | 21.16 |
| Asp227 [N] | Cys2037 [O] | 3.06 | Thr186 | 42.23 | Pro2033 | 117.32 |
| Asp227 [Oδ1] | Cys2037 [N] | 2.69 | Glu187 | 38.90 | Val2034 | 22.08 |
| Ile225 [N] | Thr2039 [O] | 2.78 | Pro188 | 43.58 | Ala2035 | 31.92 |
| Ile225 [O] | Thr2039 [N] | 3.10 | Phe201 | 26.87 | Ser2036 | 10.10 |
| Asn223 [O] | Arg2041 [N] | 2.81 | Asn205 | 34.39 | Cys2037 | 29.71 |
| Asn223 [Oδ1] | Arg2041 [N] | 2.96 | Asn223 | 75.29 | Phe2038 | 96.80 |
| Gly222 [O] | Arg2041 [NH1] | 2.87 | Met224 | 44.27 | Thr2039 | 40.02 |
| Met224 [O] | Arg2041 [NH1] | 3.34 | Ile225 | 82.05 | Arg2041 | 179.42 |
| Tyr234 [O] | Arg2041 [NH1] | 3.33 | Pro226 | 33.78 | Met2042 | 116.83 |
| Tyr234 [O] | Arg2041 [NH2] | 3.08 | Asp227 | 71.82 | Gln2046 | 42.04 |
| Ser232 [O] | Arg2041 [NH2] | 2.70 | Tyr234 | 29.46 | Gln2047 | 6.14 |
| Asn223 [Oδ1] | Met2042 [N] | 2.84 | Lys235 | 17.49 | Ile2048 | 81.38 |
| Glu187 [N] | Cys2049 [O] | 2.97 | Tyr251 | 88.03 | Cys2049 | 24.15 |
| Glu187 [O] | Cys2049 [N] | 3.25 | Ala254 | 38.63 | Leu2050 | 41.23 |
| Glu187 [N] | Asn2051 [Oδ1] | 3.53 | Tyr262 | 46.63 | Ser2052 | 39.62 |
| Pro185 [O] | Asn2051 [N] | 3.19 | Asn271 | 24.39 | Val2053 | 17.59 |
| Gly172 [N] | Val2053 [O] | 2.83 | Met273 | 35.67 | Val2054 | 57.91 |
| Ala170 [O] | Asn2055 [N] | 2.83 | Pro350 | 46.09 | Asn2055 | 85.00 |
| Gln174 [O] | Asn2055 [Nδ2] | 3.27 | Tyr390 | 33.58 | Thr2056 | 0.00 |
| Asn271 [Oδ1] | Asn2055 [Nδ2] | 2.94 | | | Ala2057 | 47.96 |
| | | | | | Leu2058 | 94.78 |
