# Supplementary material for: Structural and Functional Insights into the Malaria Parasite Moving Junction Complex
Source: PLoS Pathog. 2012 Jun 21;8(6):e1002755. doi: 10.1371/journal.ppat.1002755 (PMC3380929; doi:10.1371/journal.ppat.1002755)
Supplement: Figure S2 — Isothermal titration calorimetry of peptide R1 binding to Pf AMA1 3D7. (PPTX) [file ppat.1002755.s002.pptx]

## Slide 1
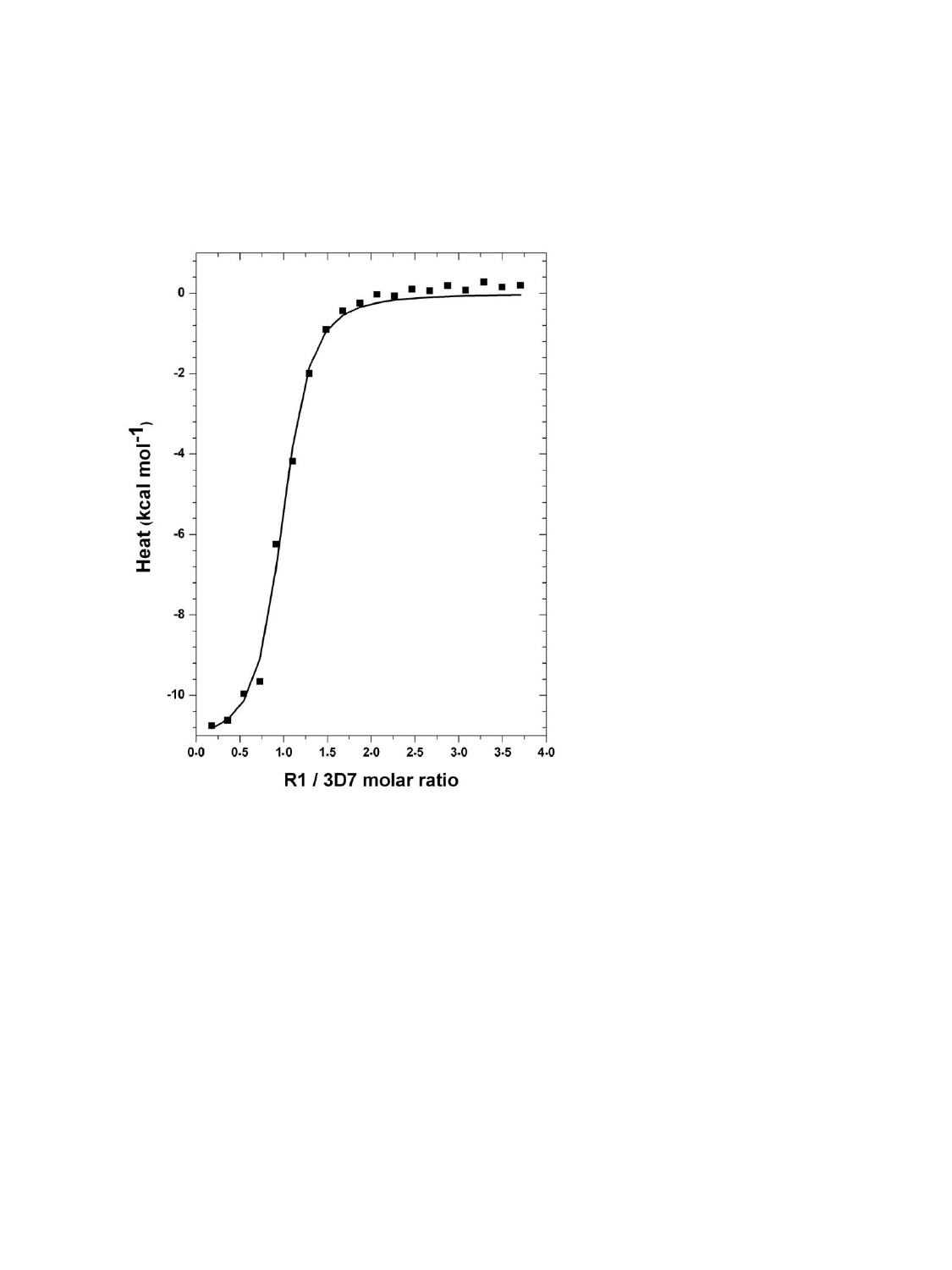

## Slide 2
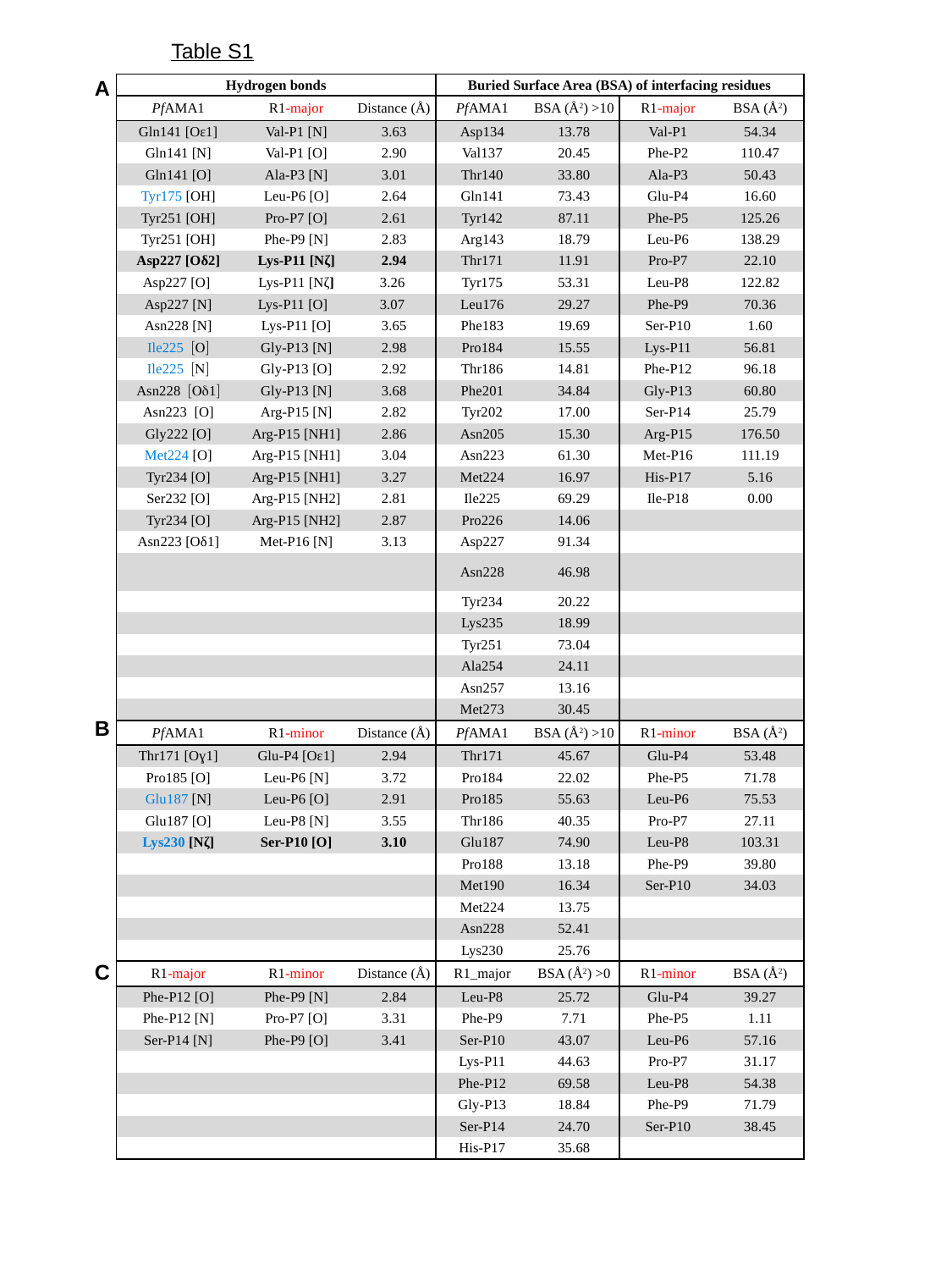

Table S1
A
| Hydrogen bonds | | | Buried Surface Area (BSA) of interfacing residues | | | |
| --- | --- | --- | --- | --- | --- | --- |
| PfAMA1 | R1-major | Distance (Å) | PfAMA1 | BSA (Å2) >10 | R1-major | BSA (Å2) |
| Gln141 [Oɛ1] | Val-P1 [N] | 3.63 | Asp134 | 13.78 | Val-P1 | 54.34 |
| Gln141 [N] | Val-P1 [O] | 2.90 | Val137 | 20.45 | Phe-P2 | 110.47 |
| Gln141 [O] | Ala-P3 [N] | 3.01 | Thr140 | 33.80 | Ala-P3 | 50.43 |
| Tyr175 [OH] | Leu-P6 [O] | 2.64 | Gln141 | 73.43 | Glu-P4 | 16.60 |
| Tyr251 [OH] | Pro-P7 [O] | 2.61 | Tyr142 | 87.11 | Phe-P5 | 125.26 |
| Tyr251 [OH] | Phe-P9 [N] | 2.83 | Arg143 | 18.79 | Leu-P6 | 138.29 |
| Asp227 [Oδ2] | Lys-P11 [Nζ] | 2.94 | Thr171 | 11.91 | Pro-P7 | 22.10 |
| Asp227 [O] | Lys-P11 [Nζ] | 3.26 | Tyr175 | 53.31 | Leu-P8 | 122.82 |
| Asp227 [N] | Lys-P11 [O] | 3.07 | Leu176 | 29.27 | Phe-P9 | 70.36 |
| Asn228 [N] | Lys-P11 [O] | 3.65 | Phe183 | 19.69 | Ser-P10 | 1.60 |
| Ile225 [O] | Gly-P13 [N] | 2.98 | Pro184 | 15.55 | Lys-P11 | 56.81 |
| Ile225 [N] | Gly-P13 [O] | 2.92 | Thr186 | 14.81 | Phe-P12 | 96.18 |
| Asn228 [Od1] | Gly-P13 [N] | 3.68 | Phe201 | 34.84 | Gly-P13 | 60.80 |
| Asn223 [O] | Arg-P15 [N] | 2.82 | Tyr202 | 17.00 | Ser-P14 | 25.79 |
| Gly222 [O] | Arg-P15 [NH1] | 2.86 | Asn205 | 15.30 | Arg-P15 | 176.50 |
| Met224 [O] | Arg-P15 [NH1] | 3.04 | Asn223 | 61.30 | Met-P16 | 111.19 |
| Tyr234 [O] | Arg-P15 [NH1] | 3.27 | Met224 | 16.97 | His-P17 | 5.16 |
| Ser232 [O] | Arg-P15 [NH2] | 2.81 | Ile225 | 69.29 | Ile-P18 | 0.00 |
| Tyr234 [O] | Arg-P15 [NH2] | 2.87 | Pro226 | 14.06 | | |
| Asn223 [Oδ1] | Met-P16 [N] | 3.13 | Asp227 | 91.34 | | |
| | | | Asn228 | 46.98 | | |
| | | | Tyr234 | 20.22 | | |
| | | | Lys235 | 18.99 | | |
| | | | Tyr251 | 73.04 | | |
| | | | Ala254 | 24.11 | | |
| | | | Asn257 | 13.16 | | |
| | | | Met273 | 30.45 | | |
| PfAMA1 | R1-minor | Distance (Å) | PfAMA1 | BSA (Å2) >10 | R1-minor | BSA (Å2) |
| Thr171 [Oɣ1] | Glu-P4 [Oɛ1] | 2.94 | Thr171 | 45.67 | Glu-P4 | 53.48 |
| Pro185 [O] | Leu-P6 [N] | 3.72 | Pro184 | 22.02 | Phe-P5 | 71.78 |
| Glu187 [N] | Leu-P6 [O] | 2.91 | Pro185 | 55.63 | Leu-P6 | 75.53 |
| Glu187 [O] | Leu-P8 [N] | 3.55 | Thr186 | 40.35 | Pro-P7 | 27.11 |
| Lys230 [Nζ] | Ser-P10 [O] | 3.10 | Glu187 | 74.90 | Leu-P8 | 103.31 |
| | | | Pro188 | 13.18 | Phe-P9 | 39.80 |
| | | | Met190 | 16.34 | Ser-P10 | 34.03 |
| | | | Met224 | 13.75 | | |
| | | | Asn228 | 52.41 | | |
| | | | Lys230 | 25.76 | | |
| R1-major | R1-minor | Distance (Å) | R1\_major | BSA (Å2) >0 | R1-minor | BSA (Å2) |
| Phe-P12 [O] | Phe-P9 [N] | 2.84 | Leu-P8 | 25.72 | Glu-P4 | 39.27 |
| Phe-P12 [N] | Pro-P7 [O] | 3.31 | Phe-P9 | 7.71 | Phe-P5 | 1.11 |
| Ser-P14 [N] | Phe-P9 [O] | 3.41 | Ser-P10 | 43.07 | Leu-P6 | 57.16 |
| | | | Lys-P11 | 44.63 | Pro-P7 | 31.17 |
| | | | Phe-P12 | 69.58 | Leu-P8 | 54.38 |
| | | | Gly-P13 | 18.84 | Phe-P9 | 71.79 |
| | | | Ser-P14 | 24.70 | Ser-P10 | 38.45 |
| | | | His-P17 | 35.68 | | |
B
C

## Slide 3
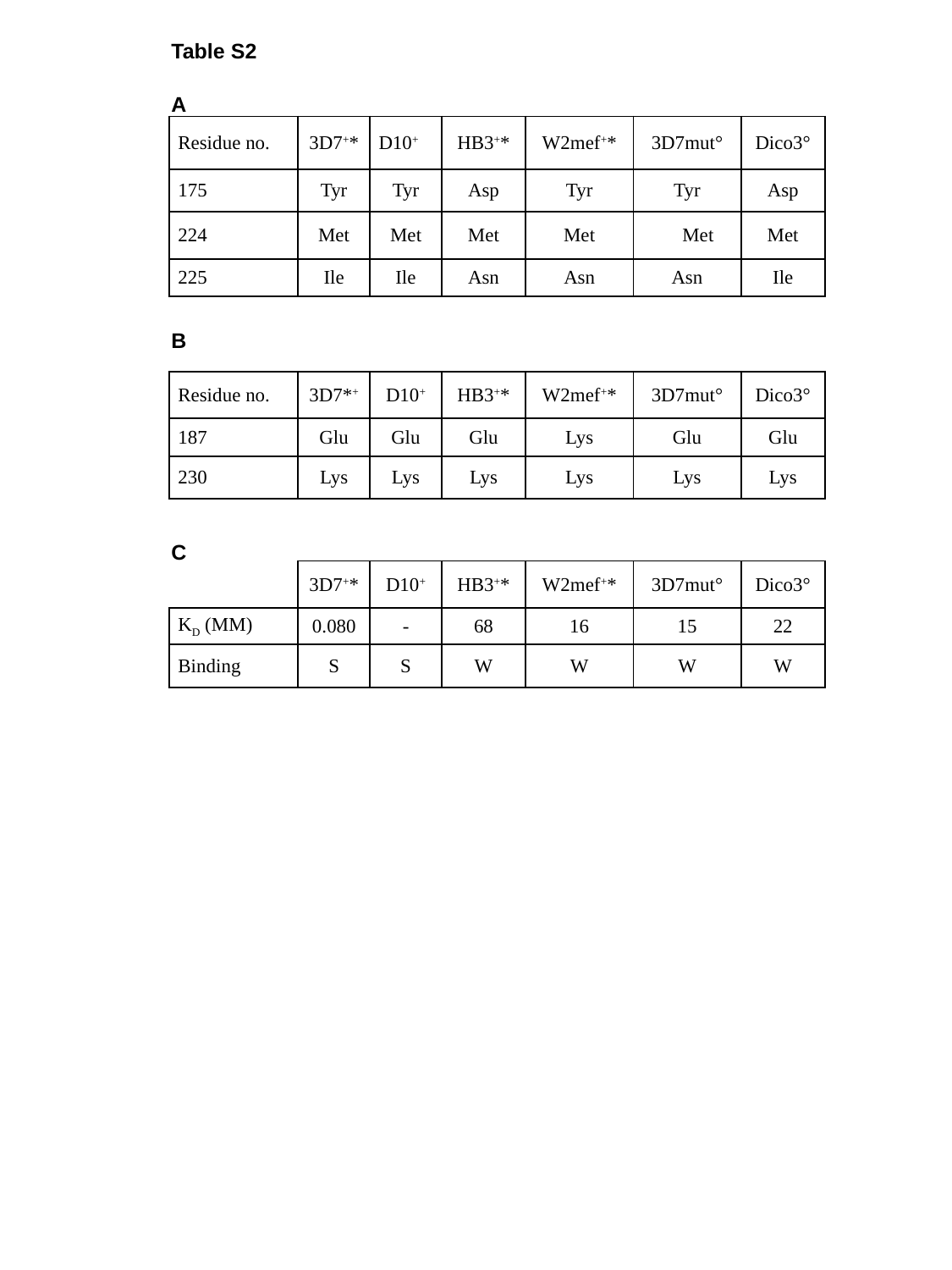

Table S2
A
| Residue no. | 3D7+\* | D10+ | HB3+\* | W2mef+\* | 3D7mut° | Dico3° |
| --- | --- | --- | --- | --- | --- | --- |
| 175 | Tyr | Tyr | Asp | Tyr | Tyr | Asp |
| 224 | Met | Met | Met | Met | Met | Met |
| 225 | Ile | Ile | Asn | Asn | Asn | Ile |
B
| Residue no. | 3D7\*+ | D10+ | HB3+\* | W2mef+\* | 3D7mut° | Dico3° |
| --- | --- | --- | --- | --- | --- | --- |
| 187 | Glu | Glu | Glu | Lys | Glu | Glu |
| 230 | Lys | Lys | Lys | Lys | Lys | Lys |
C
| | 3D7+\* | D10+ | HB3+\* | W2mef+\* | 3D7mut° | Dico3° |
| --- | --- | --- | --- | --- | --- | --- |
| KD (µM) | 0.080 | - | 68 | 16 | 15 | 22 |
| Binding | s | s | w | w | w | w |

## Slide 4
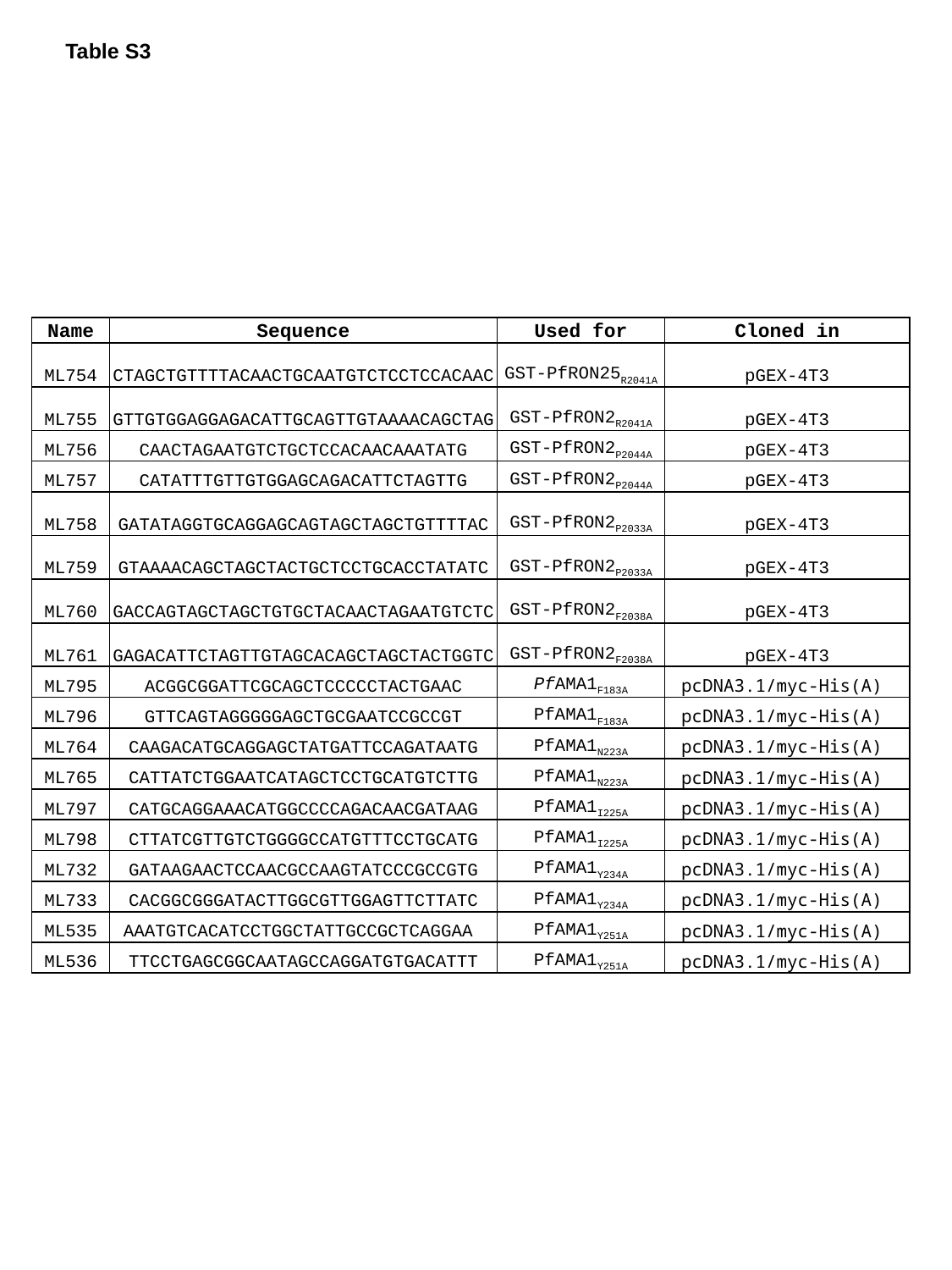

Table S3
| Name | Sequence | Used for | Cloned in |
| --- | --- | --- | --- |
| ML754 | CTAGCTGTTTTACAACTGCAATGTCTCCTCCACAAC | GST-PfRON25R2041A | pGEX-4T3 |
| ML755 | GTTGTGGAGGAGACATTGCAGTTGTAAAACAGCTAG | GST-PfRON2R2041A | pGEX-4T3 |
| ML756 | CAACTAGAATGTCTGCTCCACAACAAATATG | GST-PfRON2P2044A | pGEX-4T3 |
| ML757 | CATATTTGTTGTGGAGCAGACATTCTAGTTG | GST-PfRON2P2044A | pGEX-4T3 |
| ML758 | GATATAGGTGCAGGAGCAGTAGCTAGCTGTTTTAC | GST-PfRON2P2033A | pGEX-4T3 |
| ML759 | GTAAAACAGCTAGCTACTGCTCCTGCACCTATATC | GST-PfRON2P2033A | pGEX-4T3 |
| ML760 | GACCAGTAGCTAGCTGTGCTACAACTAGAATGTCTC | GST-PfRON2F2038A | pGEX-4T3 |
| ML761 | GAGACATTCTAGTTGTAGCACAGCTAGCTACTGGTC | GST-PfRON2F2038A | pGEX-4T3 |
| ML795 | ACGGCGGATTCGCAGCTCCCCCTACTGAAC | PfAMA1F183A | pcDNA3.1/myc-His(A) |
| ML796 | GTTCAGTAGGGGGAGCTGCGAATCCGCCGT | PfAMA1F183A | pcDNA3.1/myc-His(A) |
| ML764 | CAAGACATGCAGGAGCTATGATTCCAGATAATG | PfAMA1N223A | pcDNA3.1/myc-His(A) |
| ML765 | CATTATCTGGAATCATAGCTCCTGCATGTCTTG | PfAMA1N223A | pcDNA3.1/myc-His(A) |
| ML797 | CATGCAGGAAACATGGCCCCAGACAACGATAAG | PfAMA1I225A | pcDNA3.1/myc-His(A) |
| ML798 | CTTATCGTTGTCTGGGGCCATGTTTCCTGCATG | PfAMA1I225A | pcDNA3.1/myc-His(A) |
| ML732 | GATAAGAACTCCAACGCCAAGTATCCCGCCGTG | PfAMA1Y234A | pcDNA3.1/myc-His(A) |
| ML733 | CACGGCGGGATACTTGGCGTTGGAGTTCTTATC | PfAMA1Y234A | pcDNA3.1/myc-His(A) |
| ML535 | AAATGTCACATCCTGGCTATTGCCGCTCAGGAA | PfAMA1Y251A | pcDNA3.1/myc-His(A) |
| ML536 | TTCCTGAGCGGCAATAGCCAGGATGTGACATTT | PfAMA1Y251A | pcDNA3.1/myc-His(A) |
