# Supplementary material for: Structural and Functional Insights into the Malaria Parasite Moving Junction Complex
Source: PLoS Pathog. 2012 Jun 21;8(6):e1002755. doi: 10.1371/journal.ppat.1002755 (PMC3380929; doi:10.1371/journal.ppat.1002755)
Supplement: Table S2 — Polymorphic residues of Pf AMA1 contacting peptide R1. (A). Polymorphic residues contacting R1-major showing the sequence for strains analyzed using ELISA (*) [14], SPR (+) [13] and in this study using SPR (°). (B). Polymorphic residues contacting R1-minor, showing the sequence for strains as presented in (A). (C). Binding to PfAMA1, classified as strong (s) or weak (w) for the studies presented in (A) and (B). (PPTX) [file ppat.1002755.s004.pptx]

## Slide 1
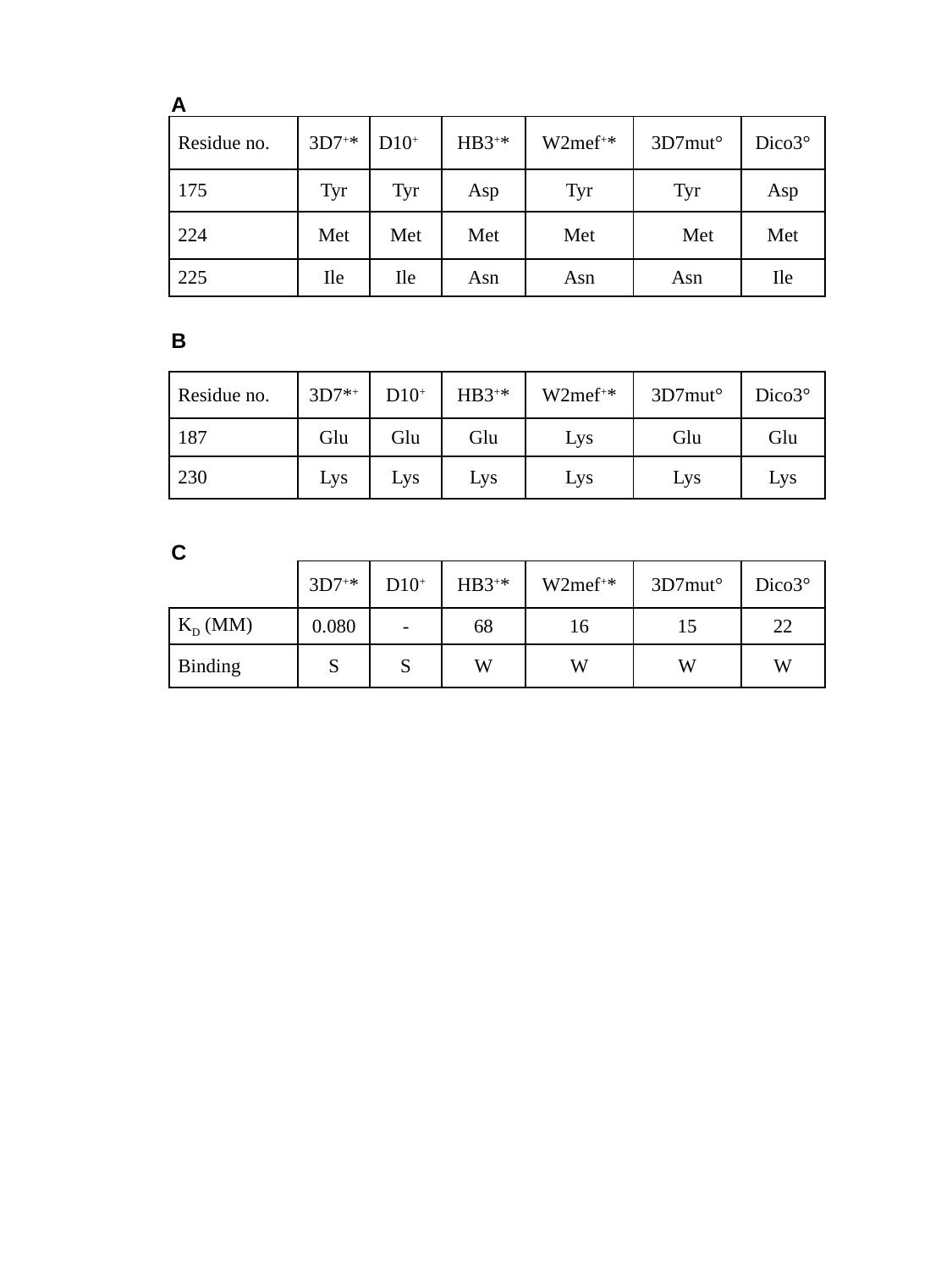

A
| Residue no. | 3D7+\* | D10+ | HB3+\* | W2mef+\* | 3D7mut° | Dico3° |
| --- | --- | --- | --- | --- | --- | --- |
| 175 | Tyr | Tyr | Asp | Tyr | Tyr | Asp |
| 224 | Met | Met | Met | Met | Met | Met |
| 225 | Ile | Ile | Asn | Asn | Asn | Ile |
B
| Residue no. | 3D7\*+ | D10+ | HB3+\* | W2mef+\* | 3D7mut° | Dico3° |
| --- | --- | --- | --- | --- | --- | --- |
| 187 | Glu | Glu | Glu | Lys | Glu | Glu |
| 230 | Lys | Lys | Lys | Lys | Lys | Lys |
C
| | 3D7+\* | D10+ | HB3+\* | W2mef+\* | 3D7mut° | Dico3° |
| --- | --- | --- | --- | --- | --- | --- |
| KD (µM) | 0.080 | - | 68 | 16 | 15 | 22 |
| Binding | s | s | w | w | w | w |

## Slide 2
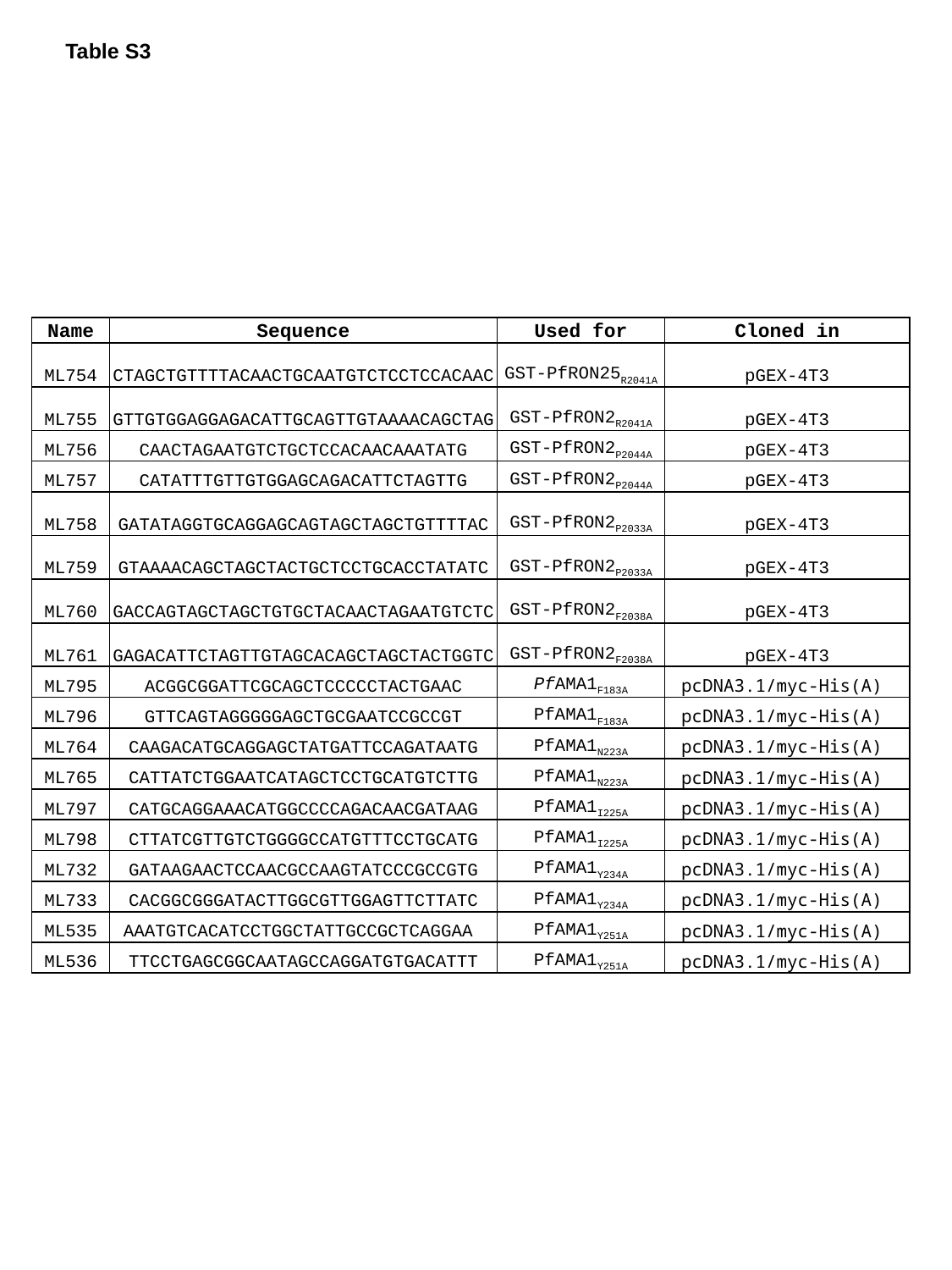

Table S3
| Name | Sequence | Used for | Cloned in |
| --- | --- | --- | --- |
| ML754 | CTAGCTGTTTTACAACTGCAATGTCTCCTCCACAAC | GST-PfRON25R2041A | pGEX-4T3 |
| ML755 | GTTGTGGAGGAGACATTGCAGTTGTAAAACAGCTAG | GST-PfRON2R2041A | pGEX-4T3 |
| ML756 | CAACTAGAATGTCTGCTCCACAACAAATATG | GST-PfRON2P2044A | pGEX-4T3 |
| ML757 | CATATTTGTTGTGGAGCAGACATTCTAGTTG | GST-PfRON2P2044A | pGEX-4T3 |
| ML758 | GATATAGGTGCAGGAGCAGTAGCTAGCTGTTTTAC | GST-PfRON2P2033A | pGEX-4T3 |
| ML759 | GTAAAACAGCTAGCTACTGCTCCTGCACCTATATC | GST-PfRON2P2033A | pGEX-4T3 |
| ML760 | GACCAGTAGCTAGCTGTGCTACAACTAGAATGTCTC | GST-PfRON2F2038A | pGEX-4T3 |
| ML761 | GAGACATTCTAGTTGTAGCACAGCTAGCTACTGGTC | GST-PfRON2F2038A | pGEX-4T3 |
| ML795 | ACGGCGGATTCGCAGCTCCCCCTACTGAAC | PfAMA1F183A | pcDNA3.1/myc-His(A) |
| ML796 | GTTCAGTAGGGGGAGCTGCGAATCCGCCGT | PfAMA1F183A | pcDNA3.1/myc-His(A) |
| ML764 | CAAGACATGCAGGAGCTATGATTCCAGATAATG | PfAMA1N223A | pcDNA3.1/myc-His(A) |
| ML765 | CATTATCTGGAATCATAGCTCCTGCATGTCTTG | PfAMA1N223A | pcDNA3.1/myc-His(A) |
| ML797 | CATGCAGGAAACATGGCCCCAGACAACGATAAG | PfAMA1I225A | pcDNA3.1/myc-His(A) |
| ML798 | CTTATCGTTGTCTGGGGCCATGTTTCCTGCATG | PfAMA1I225A | pcDNA3.1/myc-His(A) |
| ML732 | GATAAGAACTCCAACGCCAAGTATCCCGCCGTG | PfAMA1Y234A | pcDNA3.1/myc-His(A) |
| ML733 | CACGGCGGGATACTTGGCGTTGGAGTTCTTATC | PfAMA1Y234A | pcDNA3.1/myc-His(A) |
| ML535 | AAATGTCACATCCTGGCTATTGCCGCTCAGGAA | PfAMA1Y251A | pcDNA3.1/myc-His(A) |
| ML536 | TTCCTGAGCGGCAATAGCCAGGATGTGACATTT | PfAMA1Y251A | pcDNA3.1/myc-His(A) |
