# Supplementary material for: Structural and Functional Insights into the Malaria Parasite Moving Junction Complex
Source: PLoS Pathog. 2012 Jun 21;8(6):e1002755. doi: 10.1371/journal.ppat.1002755 (PMC3380929; doi:10.1371/journal.ppat.1002755)
Supplement: Table S3 — Primers used in this study. (PPTX) [file ppat.1002755.s005.pptx]

## Slide 1
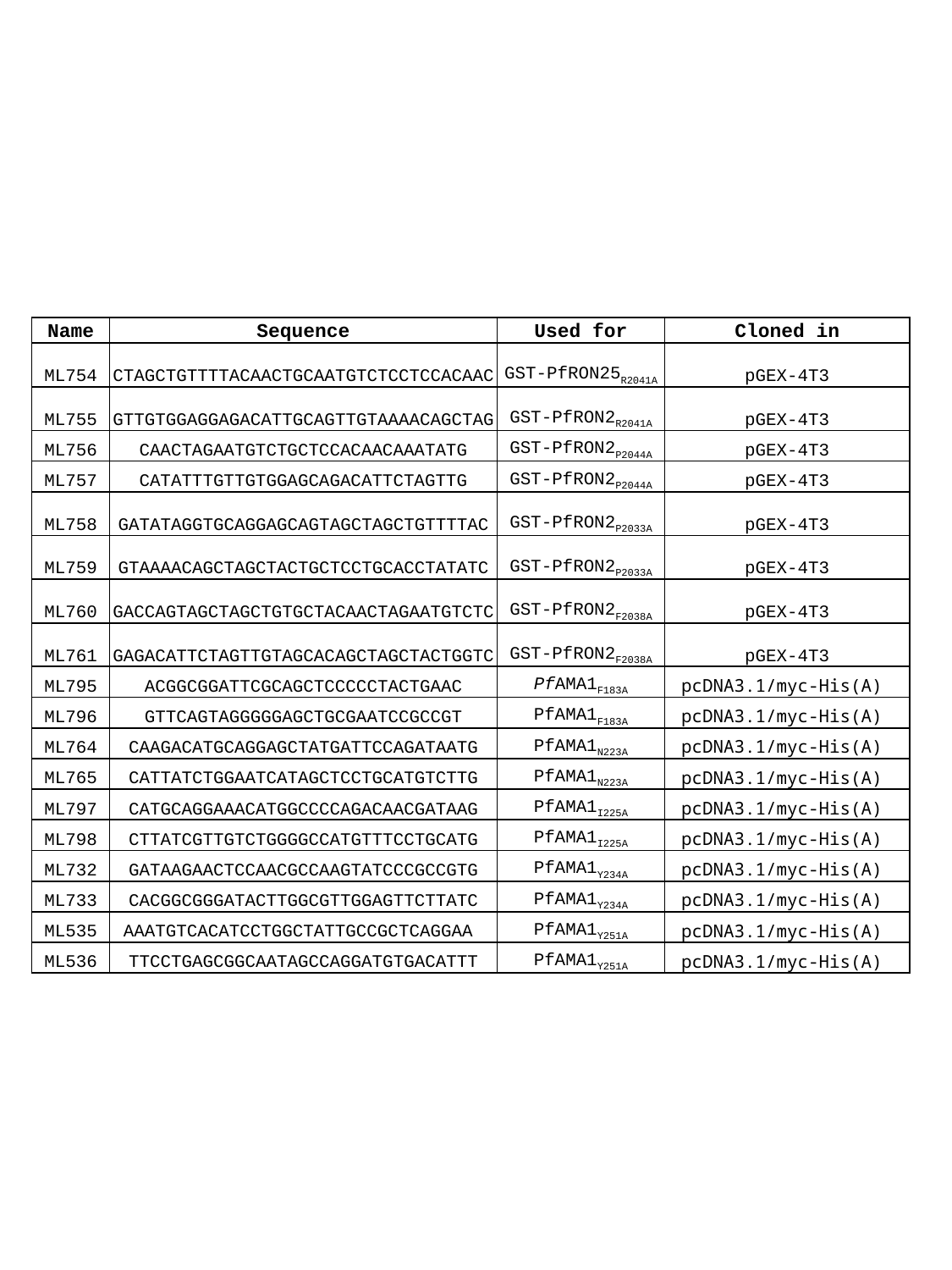

| Name | Sequence | Used for | Cloned in |
| --- | --- | --- | --- |
| ML754 | CTAGCTGTTTTACAACTGCAATGTCTCCTCCACAAC | GST-PfRON25R2041A | pGEX-4T3 |
| ML755 | GTTGTGGAGGAGACATTGCAGTTGTAAAACAGCTAG | GST-PfRON2R2041A | pGEX-4T3 |
| ML756 | CAACTAGAATGTCTGCTCCACAACAAATATG | GST-PfRON2P2044A | pGEX-4T3 |
| ML757 | CATATTTGTTGTGGAGCAGACATTCTAGTTG | GST-PfRON2P2044A | pGEX-4T3 |
| ML758 | GATATAGGTGCAGGAGCAGTAGCTAGCTGTTTTAC | GST-PfRON2P2033A | pGEX-4T3 |
| ML759 | GTAAAACAGCTAGCTACTGCTCCTGCACCTATATC | GST-PfRON2P2033A | pGEX-4T3 |
| ML760 | GACCAGTAGCTAGCTGTGCTACAACTAGAATGTCTC | GST-PfRON2F2038A | pGEX-4T3 |
| ML761 | GAGACATTCTAGTTGTAGCACAGCTAGCTACTGGTC | GST-PfRON2F2038A | pGEX-4T3 |
| ML795 | ACGGCGGATTCGCAGCTCCCCCTACTGAAC | PfAMA1F183A | pcDNA3.1/myc-His(A) |
| ML796 | GTTCAGTAGGGGGAGCTGCGAATCCGCCGT | PfAMA1F183A | pcDNA3.1/myc-His(A) |
| ML764 | CAAGACATGCAGGAGCTATGATTCCAGATAATG | PfAMA1N223A | pcDNA3.1/myc-His(A) |
| ML765 | CATTATCTGGAATCATAGCTCCTGCATGTCTTG | PfAMA1N223A | pcDNA3.1/myc-His(A) |
| ML797 | CATGCAGGAAACATGGCCCCAGACAACGATAAG | PfAMA1I225A | pcDNA3.1/myc-His(A) |
| ML798 | CTTATCGTTGTCTGGGGCCATGTTTCCTGCATG | PfAMA1I225A | pcDNA3.1/myc-His(A) |
| ML732 | GATAAGAACTCCAACGCCAAGTATCCCGCCGTG | PfAMA1Y234A | pcDNA3.1/myc-His(A) |
| ML733 | CACGGCGGGATACTTGGCGTTGGAGTTCTTATC | PfAMA1Y234A | pcDNA3.1/myc-His(A) |
| ML535 | AAATGTCACATCCTGGCTATTGCCGCTCAGGAA | PfAMA1Y251A | pcDNA3.1/myc-His(A) |
| ML536 | TTCCTGAGCGGCAATAGCCAGGATGTGACATTT | PfAMA1Y251A | pcDNA3.1/myc-His(A) |
